# The potential and therapeutic advances of the integrin family in neurological disorders

**DOI:** 10.4103/NRR.NRR-D-25-00020

**Published:** 2025-08-13

**Authors:** Xingfang Zhang, Liang Gao, Yiwen Wang, Qian Meng, Min Bai, Dong Xu, Yanhua Wang, Jianv Wang, Hongtao Bi, Yi Ding

**Affiliations:** 1 Qinghai Provincial Key Laboratory of Tibetan Medicine Pharmacology and Safety Evaluation, Northwest Institute of Plateau Biology, Chinese Academy of Science, Xining, Qinghai Province, China; 2 Department of Pharmacy, Xijing Hospital, Fourth Military Medical University, Xi’an, Shaanxi Province, China; 3 Department of Pharmacology, Shaanxi University of Traditional Chinese Medicine, Xianyang, Shaanxi Province, China; 4 College of Life Sciences, Northwest University, Xi’an, Shaanxi Province, China

**Keywords:** Alzheimer’s disease, blood–brain barrier, central nervous system, extracellular matrix, hemorrhagic stroke, integrin, ischemic stroke, multiple sclerosis, neurological disorders, Parkinson’s disease

## Abstract

Neurological disorders encompass a diverse and heterogeneous group of medical conditions, including cerebrovascular diseases (e.g., stroke), neurodegenerative diseases (e.g., Alzheimer’s disease and Parkinson’s disease), and autoimmune demyelinating disorders (e.g., multiple sclerosis). With the global aging population, the incidence of these disorders continues to rise, posing significant challenges to healthcare systems and socio-economic structures. Recent studies have highlighted integrins—a family of transmembrane glycoprotein receptors—as critical regulators of central nervous system function, making them a focal point in neurological disease research. By interacting with the extracellular matrix, integrins modulate cell adhesion, signal transduction, and inflammatory responses, playing indispensable roles in neuronal development, synaptic plasticity, and blood–brain barrier maintenance. Dysregulated integrin signaling has been implicated in the pathophysiology of various neurological disorders, suggesting that integrin-targeting interventions, including integrin antagonists or agonists, could represent novel therapeutic strategies. Preclinical and clinical studies have demonstrated that modulating integrin function influences disease progression, offering promising avenues for the development of precision medicine approaches. This review provides a comprehensive analysis of integrin structure, classification, and their physiological and pathological roles in the central nervous system, with a focus on their molecular mechanisms in neurological disorders. Furthermore, we evaluate the therapeutic potential and challenges associated with integrin-targeted interventions. By elucidating the mechanistic underpinnings of integrin function in the central nervous system, this review aims to advance our understanding of their translational potential, laying the groundwork for the development of innovative therapeutic strategies.

## Introduction

Neurological disorders represent a heterogeneous group of medical conditions that affect both the central nervous system (CNS), comprising the brain and spinal cord, and the peripheral nervous system distributed throughout the body. This broad disease category is characterized by diverse etiologies, ranging from genetic predisposition to environmental triggers, and encompasses a spectrum of pathologies that can manifest as either acute or chronic conditions (Gadhave et al., 2024). Cerebrovascular diseases, such as ischemic stroke (IS), which results from arterial occlusion impairing cerebral perfusion, and hemorrhagic stroke (HS), caused by vascular rupture and subsequent intracerebral hemorrhage, are among the most critical conditions within this category (Wen et al., 2024; Lui et al., 2025). Neurodegenerative diseases, including Alzheimer’s disease (AD), marked by progressive cognitive decline and memory impairment (Amini et al., 2024; Jia et al., 2024), and Parkinson’s disease (PD), characterized by motor dysfunctions such as tremors and rigidity, further illustrate the complexity of these disorders (Calabrese et al., 2024; Sharma and Mittal, 2024). Autoimmune demyelinating diseases, exemplified by multiple sclerosis (MS), arise from aberrant immune attacks on the protective myelin sheath of neurons, disrupting neuronal communication (Madipally and Pathi, 2024; Mohammad, 2024). Additionally, traumatic injuries to the CNS, whether due to accidents or other physical insults, add another layer of complexity to this diverse spectrum of neurological conditions (Grinin et al., 2024; Ogugua et al., 2024; Rakshe et al., 2024). The rising prevalence of neurological disorders is intricately linked to global demographic shifts, particularly the aging population (Cui et al., 2024b; Grotewold and Albin, 2024; Prust et al., 2024). As life expectancy increases, the incidence of age-related neurological diseases escalates correspondingly, imposing significant burdens on healthcare resources and socio-economic structures (Chockalingam et al., 2024; Sen and Nagaram, 2024). Stroke serves as a prominent example of this trend, affecting individuals across all age groups but with a pronounced increase in incidence among the elderly (Ibeneme et al., 2024; Zhu et al., 2024). It remains a leading cause of morbidity and mortality worldwide, with the Global Burden of Disease study estimating approximately 12.2 million new stroke cases annually (Prust et al., 2024; Reis Júnior et al., 2024). AD, the predominant cause of dementia, represents another facet of this growing public health crisis, accounting for nearly 70% of all dementia cases worldwide. Projections indicate that by the mid-21^st^ century, the number of individuals affected by AD will surge to 150 million (Scheltens et al., 2021). Although less prevalent than AD, PD remains a formidable neurodegenerative disorder, profoundly impacting motor function and overall quality of life (Kilinc et al., 2024; Tedeschi et al., 2024). Its management necessitates complex medical interventions and long-term care, placing substantial strain on healthcare systems. MS, a chronic autoimmune disorder, commonly manifests during early adulthood, leading to a constellation of physical and cognitive impairments that further complicate the landscape of neurological diseases (Feinstein, 2022; Marrie et al., 2022; Kumar et al., 2023). The chronic nature of MS, coupled with its onset during a relatively young age, means that affected individuals may require decades of disease management, exerting profound personal and societal implications. These neurological disorders, despite their distinct pathophysiologies, share common molecular and cellular mechanisms, underscoring the urgency for novel therapeutic strategies to mitigate their devastating impact.

**Figure 1:**
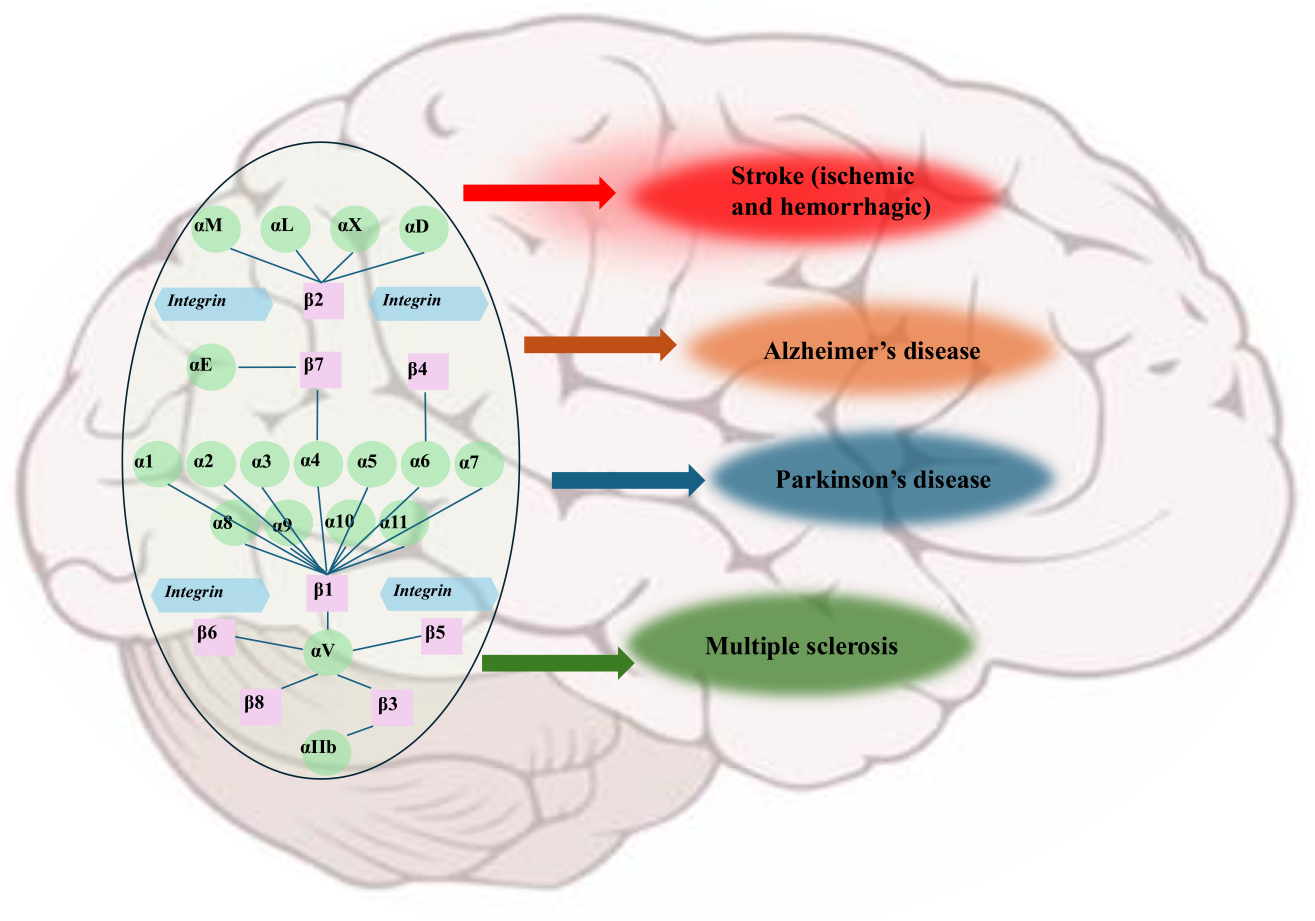
Integrin species and their association with central nervous system disorders. On the left side of the figure, various integrin subunits are listed, including multiple members of the α and β families. Each integrin is non-covalently composed of α and β subunits, and their connections to corresponding diseases are indicated, suggesting the potential roles integrins may play in these neurodegenerative diseases and highlighting their possibility as potential therapeutic targets.

Integrins are a class of heterodimeric transmembrane glycoprotein receptors that primarily function as signaling proteins in mammals, playing a pivotal role in mediating interactions between cells and the extracellular matrix (ECM) (Cui et al., 2024a; Zhang et al., 2024a). Given their critical involvement in various cellular processes, integrins have garnered significant attention as potential therapeutic targets for multiple CNS disorders (Tvaroška et al., 2023; Zhang et al., 2023a). The integrin family comprises a set of non-covalently linked heterodimeric cell surface receptors, each consisting of an α and a β subunit. In mammals, 18 α subunits (including ITGA1 (α1), ITGA2 (α2), ITGA3 (α3), ITGA4 (α4), ITGA5 (α5), ITGA6 (α6), ITGA7 (α7), ITGA8 (α8), ITGA9 (α9), ITGA10 (α10), ITGA11 (α11), ITGAV (αV), ITGAE (αE), ITGAM (αM), ITGAX (αX), ITGAD (αD), ITGAL (αL), ITGAIIB (αIIb)) and 8 β subunits (ranging from β1 to β8, including ITGB1 (β1), ITGB2 (β2), ITGB3 (β3), ITGB4 (β4), ITGB5 (β5), ITGB6 (β6), ITGB7 (β7), ITGB8 (β8)) can combinatorially assemble into 24 distinct αβ heterodimers (**Figure 1**), each exhibiting specific affinity for particular ECM components or ligands (Kanchanawong and Calderwood, 2023; He et al., 2024b). This structural diversity enables integrins to interact with a wide range of ECM proteins, including fibronectin, laminin, collagen, and vitronectin, thereby mediating cell-ECM adhesion and bidirectional signal transduction. From an evolutionary perspective, integrins are highly conserved across metazoan species, underscoring their fundamental role in multicellular development and homeostasis. Studies indicate that integrin subunits have diversified to fulfill species-specific functions while maintaining the core structural and functional motifs essential for ligand binding and signal transduction (Takagi and Springer, 2002). This evolutionary conservation highlights the crucial role of integrins in the structural integrity of tissues and intercellular communication.

Beyond their structural diversity, integrins exhibit significant functional variability. Upon ligand binding, each integrin heterodimer can initiate distinct intracellular signaling cascades, activating pathways such as focal adhesion kinase (FAK), integrin-linked kinase (ILK), and mitogen-activated protein kinase (MAPK) (Pang et al., 2023b). These signaling events are essential for transmitting both mechanical and biochemical cues from the ECM into the cell, thereby influencing cellular responses to environmental stimuli. Moreover, integrins are implicated in a myriad of physiological and pathological processes, including angiogenesis, wound healing, immune responses, and cancer metastasis (Kanchanawong et al., 2010). Their dynamic regulation and multifunctionality underscore their significance in both normal cellular function and disease pathogenesis, positioning integrins as promising targets for therapeutic intervention across a spectrum of CNS disorders.

In a healthy brain, integrins play a pivotal role in neuronal development, contributing to processes such as neuronal migration, axon guidance, synapse formation, and synaptic plasticity (Davis-Lunn et al., 2023; Minegishi et al., 2023). Beyond neurons, integrins also regulate interactions between astrocytic endfeet and microvasculature, influence the migration of oligodendrocyte precursor cells (OPCs), and modulate microglial activation, collectively promoting neurovascular function and homeostasis (Nasu-Tada et al., 2005; Lathia et al., 2008; Zhao et al., 2009). A growing body of evidence underscores that dysregulated integrin signaling can initiate or exacerbate pathological processes in neurological disorders (Cui et al., 2024a). For instance, in IS, leukocyte-specific integrins—such as β2 integrins—are crucial mediators of immune cell adhesion and infiltration across the blood–brain barrier (BBB), exacerbating neuroinflammation and tissue damage (Iadecola and Anrather, 2011). In AD, specific integrin subtypes, including αVβ3 and αMβ2 (CD11b/CD18, Mac-1), influence microglial phagocytosis of amyloid-β (Aβ) and modulate the progression of neuroinflammatory cascades surrounding amyloid plaques (Wen and Zhang, 2023; Ivanova et al., 2024; Klaus et al., 2024). Studies on PD indicate that integrin-mediated glial responses contribute to dopaminergic neuronal survival, highlighting the role of integrin-ligand interactions in neuroinflammation and neuroprotection (Bao et al., 2024; Huang et al., 2025). In MS, α4 integrin (α4β1) has been firmly established as a key mediator of T lymphocyte extravasation into the CNS (Rice et al., 2005; Steinman, 2009). Natalizumab, a monoclonal antibody (mAb) targeting α4 integrin, has demonstrated therapeutic efficacy in blocking integrin signaling within the autoimmune demyelinating environment (Frisch, 2024; Gold et al., 2024). Thus, targeting integrins presents a compelling opportunity to modulate multiple pathological pathways, including inflammation, apoptosis, autophagy, oxidative stress, and BBB dysfunction. This multifaceted approach aligns with the current understanding that most neurological disorders involve complex, overlapping mechanisms. For example, integrin antagonists could mitigate leukocyte infiltration and subsequent inflammatory damage in stroke, whereas integrin agonists or activators may enhance neuroprotection or promote the phagocytic clearance of toxic protein aggregates in AD and PD (Ou et al., 2021; Zhang et al., 2023b; Neili et al., 2024). Consequently, integrin-targeted strategies offer a promising avenue for addressing both acute CNS injuries and chronic neurodegenerative diseases.

Given the growing recognition of integrin biology and its relevance to the pathophysiology of CNS disorders, this review aims to provide an in-depth analysis of the roles of integrin family members across various neurological diseases. We begin by summarizing our literature search strategy, detailing the databases and keywords employed to comprehensively cover integrin-related research in neuroscience. Next, we delve into the structural and classificatory aspects of integrins, emphasizing how distinct α and β subunits confer specificity for ECM ligands and intracellular signaling pathways. Building upon this foundation, we examine the physiological distribution and functions of integrins in the healthy CNS, highlighting their roles in neurodevelopment and homeostasis. We then explore the mechanistic underpinnings of integrin dysregulation in neurological diseases, with a particular focus on stroke (ischemic and hemorrhagic), AD, PD, and MS. In doing so, we illustrate how integrin-mediated processes influence inflammation, cell survival, angiogenesis, immune cell trafficking, and synaptic integrity. Subsequently, we investigate the therapeutic landscape, summarizing preclinical and clinical trials targeting integrins in these conditions, while critically evaluating the opportunities and challenges associated with translating such interventions into clinical practice. Finally, we outline the current limitations in integrin research—including heterogeneity in animal models, off-target effects, and compensatory signaling—and propose future directions that may accelerate the development of integrin-based therapeutic strategies for neurological disorders. By offering a comprehensive perspective on the structure-function relationships of integrins and their broad impact on brain pathophysiology, this review aspires to serve as an authoritative resource for researchers, clinicians, and drug developers. A deeper understanding of integrin signaling in the CNS holds the potential to drive innovative strategies for the prevention, management, and possible reversal of debilitating neurological diseases.

## Search Strategy

We conducted a literature search using the primary keywords “neurological disorders,” “stroke,” “ischemic stroke,” “hemorrhagic stroke,” “Alzheimer’s disease,” “Parkinson’s disease,” and “multiple sclerosis” (Keyword Set 1), combined with “integrins,” “ITGA,” and “ITGB” (Keyword Set 2), and “inflammation,” “cell survival,” “angiogenesis,” “immune cell trafficking,” “blood–brain barrier,” and “synaptic integrity” (Keyword Set 3). To broaden the search scope, we incorporated synonyms and Medical Subject Headings (MeSH) terms. The databases searched included Google Scholar, PubMed, ScienceDirect, Medline, Embase, and Web of Science. The time range of the search was from the 1980s to the 2020s. Retrieved citations were systematically reviewed to identify articles relevant to this study. Duplicate and irrelevant studies were excluded. The search and screening process were conducted independently and replicated to ensure rigor and reproducibility.

## Research Trajectory of Integrins

Integrins are a class of crucial cell surface receptors that play essential roles in mediating interactions between cells and the ECM, as well as between adjacent cells. Since their discovery in the early 1980s, the research on integrin family members has progressed through several key stages, spanning from basic biology to clinical applications across a broad range of fields. In 1987, Richard O. Hynes and Erkki Ruoslahti independently and nearly simultaneously identified these transmembrane receptor proteins. Hynes (1987) coined the term “integrin,” emphasizing its role in “integrating” cellular interactions with the ECM, while Ruoslahti and Pierschbacher (1987) through his studies on fibronectin-cell interactions, revealed integrins’ pivotal role in cell adhesion. That same year, scientists successfully cloned the β subunits of integrins and discovered that integrins are heterodimeric receptors composed of these two subunits. This breakthrough laid the foundation for further investigations into the structure and function of integrins (Hemler et al., 1987; Kishimoto et al., 1987). Subsequently, researchers also demonstrated that integrins mediate cell adhesion, migration, and signal transduction through their interactions with ECM proteins such as fibronectin and laminin (Staunton et al., 1988; Ignotz et al., 1989). As research advanced into the 1990s, the understanding of integrins’ structural and functional roles accelerated. In 1994, the structure of the integrin αIIbβ3 was elucidated, revealing the conformational changes of integrins on the cell membrane and their ligand-binding mechanisms, providing crucial insights into how these conformational shifts regulate integrin function (Calvete, 1994). That same year, the first integrin-targeting drug, abciximab, was approved by the U.S. Food and Drug Administration for the prevention of thrombus formation after percutaneous coronary interventions. Abciximab, a mAb against integrin αIIbβ3, works by inhibiting platelet aggregation, reducing thrombotic risks, and marking a significant breakthrough in the clinical application of integrin research (Massé et al., 1999). By 1995, it became evident that integrins were not only involved in cell adhesion but also played a significant role in intracellular signaling. Through interactions with cytoskeletal proteins such as talin and vinculin, integrins relay extracellular signals to the interior of the cell, regulating cell proliferation, differentiation, and survival. This discovery underscored the multifunctionality of integrins in cellular biology (Bates et al., 1995; Yamada and Miyamoto, 1995). In 1998, integrins were found to be critical in tumor metastasis and angiogenesis. Through interactions with molecules, such as vascular endothelial growth factor (VEGF), integrins promote the formation of tumor vasculature, supporting tumor growth and dissemination, and providing novel targets for anti-cancer drug development (Clezardin, 1998).

In the early 2000s, integrin research further shifted from fundamental science to clinical applications. In 2000, integrins were implicated in osteoporosis, with integrin αvβ3 shown to be important in osteoclast function (Teitelbaum, 2000). In 2002, researchers identified the critical role of integrins in inflammation, where they mediate leukocyte migration to sites of inflammation through interactions with surface ligands on white blood cells (Krieglstein et al., 2002), opening new avenues for the development of anti-inflammatory drugs. In 2004, the α4 integrin inhibitor Natalizumab was granted Fast Track designation by the U.S. Food and Drug Administration as a novel biologic agent for the treatment of multiple sclerosis and Crohn’s disease. In November of the same year, it received conditional approval for market release prior to the completion of Phase III clinical trials. However, due to reports of a rare but severe adverse event—progressive multifocal leukoencephalopathy—the drug was withdrawn from the market in 2005. Given the urgent therapeutic demand, the U.S. Food and Drug Administration approved its reintroduction in June 2006 under a risk minimization plan known as the TOUCH program. In the 2010s, integrin research further expanded into immunotherapy and regenerative medicine. In 2011, a study revealed that integrins play an important role in regulating T cell function (Worthington et al., 2011). By binding to ligands on T cell surfaces, integrins control T cell activation, migration, and effector functions, thus opening new opportunities for integrin-based immunotherapies. In 2013, integrins were found to be pivotal in stem cell differentiation and tissue regeneration (Brafman et al., 2013). By interacting with the ECM, integrins regulate stem cell self-renewal and differentiation, promoting tissue repair and regeneration, and providing a theoretical foundation for regenerative medicine. In 2014, the first mAb targeting integrin α4β7, Vedolizumab, was approved by the U.S. Food and Drug Administration for the treatment of inflammatory bowel disease. Vedolizumab works by inhibiting the interaction between integrin α4β7 and intestinal endothelial cells, thereby reducing inflammatory responses and further solidifying the importance of integrins in immune regulation (Amiot and Peyrin-Biroulet, 2015). In 2018, scientists employed cryo-electron microscopy to reveal the high-resolution structure of integrin αvβ8, providing molecular insights into its ligand-binding mechanism and laying the groundwork for the development of integrin αvβ8-targeting therapeutics (Cormier et al., 2018). As we entered the 2020s, integrin research continued to progress. In 2021, integrins were implicated in the pathogenesis of coronavirus disease 2019, with integrin’s role in facilitating severe acute respiratory syndrome coronavirus-2 entry into host cells, offering new targets for antiviral drug development (Dakal, 2021). In 2022, a series of significant breakthroughs in the field of integrin-targeted therapies were achieved. These include the successful Phase III clinical trial of the ^99m^Tc-HYNIC-PEG4-E[PEG4-c(RGDfK)]_2_ imaging agent, the approval of Carotegrast, the first oral integrin-targeted drug, by Japan’s Pharmaceuticals and Medical Devices Agency, and the promising safety and efficacy data from the Phase IIa clinical trial of the oral integrin αvβ6/αvβ1 antagonist PLN-74809 in patients with idiopathic pulmonary fibrosis. These advancements underscore the transformative potential of integrin-targeted therapies in expanding market opportunities.

In conclusion, research on the integrin family has progressed from fundamental biological discoveries to clinical applications since its inception in the 1980s. From early structural elucidation to understanding its role in diseases and drug development, integrin research has driven scientific and medical progress. With continuing advancements in technology, integrins are poised to play an even greater role in immunotherapy, regenerative medicine, and gene therapy. The study of integrins has not only deepened our understanding of cellular biology and disease mechanisms but also provided crucial scientific groundwork for the development of novel therapeutic strategies (**Additional Table 1**).

**Additional Table 1:** The research trajectory of integrins

Time	Events
1987	Richard O. Hynes and Erkki Ruoslahti independently and nearly simultaneously identified these transmembrane receptor proteins. Hynes coined the term "integrin," emphasizing its role in "integrating" cellular interactions with the ECM, while Ruoslahti, through his studies on fibronectin-cell interactions, revealed integrins' pivotal role in cell adhesion; scientists successfully cloned the β subunits of integrins and discovered that integrins are heterodimeric receptors composed of these two subunits.
1989	Researchers demonstrated that integrins mediate cell adhesion, migration, and signal transduction through their interactions with ECM proteins such as fibronectin and laminin.
1994	The structure of the integrin aIIbβ3 was elucidated, revealing the conformational changes of integrins on the cell membrane and their ligand-binding mechanisms; the first integrin aIIbβ3-targeting drug, abciximab, was approved by the FDA for the prevention of thrombus formation after percutaneous coronary interventions.
1995	It became evident that integrins were not only involved in cell adhesion but also played a significant role in intracellular signaling. Through interactions with cytoskeletal proteins such as talin and vinculin, integrins relay extracellular signals to the interior of the cell, regulating cell proliferation, differentiation, and survival.
1998	Integrins were found to be critical in tumor metastasis and angiogenesis. Through interactions with molecules like vascular endothelial growth factor, integrins promote the formation of tumor vasculature, supporting tumor growth and dissemination, and providing novel targets for anti-cancer drug development
2000	Integrins were implicated in osteoporosis, with integrin αvβ3 shown to be important in osteoclast function.
2002	Researchers identified the critical role of integrins in inflammation, where they mediate leukocyte migration to sites of inflammation through interactions with surface ligands on white blood cells.
2004	In 2004, the α4 integrin inhibitor Natalizumab was granted Fast Track designation by the FDA as a novel biologic agent for the treatment of multiple sclerosis and Crohn's
2006	disease. In November of the same year, it received conditional approval for market release prior to the completion of Phase III clinical trials. However, due to reports of a rare but severe adverse event-progressive multifocal leukoencephalopathy—the drug was withdrawn from the market in 2005. Given the urgent therapeutic demand, the FDA approved its reintroduction in June 2006 under a risk minimization plan known as the TOUCH program.
2011	Studies revealed that integrins play an important role in regulating T cell function.
2013	Integrins were found to be pivotal in stem cell differentiation and tissue regeneration.
2014	The first monoclonal antibody targeting integrin α4β7, Vedolizumab, was approved by the FDA for the treatment of inflammatory bowel disease.
2018	Scientists employed cryo-electron microscopy to reveal the high-resolution structure of integrin αvβ8, providing molecular insights into its ligand-binding mechanism and laying the groundwork for the development of integrin αvβ8-targeting therapeutics
2021	Integrins were implicated in the pathogenesis of coronavirus disease 2019, with integrin's role in facilitating severe acute respiratory syndrome coronavirus-2 entry into host cells, offering new targets for antiviral drug development.
2022	The successful Phase III clinical trial of the ^99m^Tc-HYNIC-PEG4-E[PEG4-c(RGDfK)]2 imaging agent, the approval of Carotegrast, the first oral integrin-targeted drug, by Japan's Pharmaceuticals and Medical Devices Agency, and the promising safety and efficacy data from the Phase IIa clinical trial of the oral integrin αvβ6/αvβ1 antagonist PLN- 74809 in patients with idiopathic pulmonary fibrosis.

ECM: Extracellular matrix; FDA: U.S. Food and Drug Administration.

## Structure, Classification, and Function of Integrins

### Structure and classification of integrins

Each integrin subunit consists of three major components: the extracellular region, a single transmembrane domain, and a short cytoplasmic tail. In the extracellular region, the α subunit typically contains a seven-bladed β-propeller structure, and in most cases (except for the “αI-less” family), it also includes an inserted αI domain capable of directly binding specific ECM ligands (Xiong et al., 2001; Mould and Humphries, 2004). In contrast, the β subunit comprises an I-like domain, four epidermal growth factor-like repeats, and a β-tail domain, with the I-like domain playing a critical role in cation coordination and determining ligand specificity (Xiong et al., 2001). Next, the single transmembrane domain is characterized by the close proximity of α and β transmembrane helices, which are stabilized through specific salt bridges or hydrogen bonds, a structural arrangement that helps maintain the inactive conformation of the integrin (Luo et al., 2007; Springer and Dustin, 2012). Finally, the short cytoplasmic tail, although relatively brief (around 20 to 70 amino acids in length) (Takada et al., 2007), is capable of recruiting or interacting with numerous cytoskeletal and signaling proteins, such as talin, kindlin, and paxillin. These interactions are crucial for the assembly of focal adhesion complexes and downstream signaling events (Calderwood et al., 2013). This modular structure not only underpins the exceptional multifunctionality of integrins but also forms the basis for their bidirectional signaling capacity, often described as “outside-in” and “inside-out” signaling mechanisms.

Integrins can be classified based on their primary ECM ligands or specific counter-receptors on the cell surface. This classification is particularly relevant in the physiological and pathological contexts of the nervous system, as integrins mediate diverse interactions with collagen, laminin, fibronectin, and other matrix components in the brain parenchyma and vasculature. Collagen-binding integrins, such as α1β1, α2β1, α10β1, and α11β1, anchor cells (e.g., astrocytes, pericytes, and endothelial cells) to the collagen matrix, contributing to basement membrane integrity and vascular stability while promoting cell migration and structural support within the CNS (Zeltz and Gullberg, 2016). Integrins that recognize the Arg-Gly-Asp (RGD) sequence, including α5β1, αVβ1, αVβ3, αVβ5, αVβ6, αVβ8, and αIIbβ3, are critical regulators of angiogenesis, wound healing, and vascular permeability (Ruoslahti, 1996). Among these, αV integrins play a key role in maintaining BBB integrity and mediating cellular responses to injury or inflammation (Hood and Cheresh, 2002). Laminin-binding integrins, such as α3β1, α6β1, α7β1, and α6β4, influence neurite outgrowth, synapse formation, and the establishment of the glial limitans, and they contribute to scaffolding for radial glial cells and the migration of neural progenitor cells (Dulabon et al., 2000). Leukocyte-specific integrins, including αLβ2, αMβ2, αXβ2, and αDβ2 (also known as lymphocyte function-associated antigen-1, Mac-1, p150,95, and CD11d/CD18, respectively), are primarily expressed on leukocytes and regulate immune cell adhesion, extravasation, and activation (Vestweber, 2015). In the CNS, dysregulated leukocyte integrin activity may drive pathological inflammation (Engelhardt and Liebner, 2014). Targeting these integrins offers a promising strategy to modulate immune responses, making them central to therapeutic approaches for inflammatory CNS diseases. Its specific ligand/anti-receptor and function are shown in **Additional Table 2**.

**Additional Table 2:** Classification of integrins and their functions

Integrin	β-Subunit	Primary ligand/anti-receptor	Function
α1β1	β1	Collagen (type I, II, IN)	Cell adhesion to collagen; Cell migration; Tissue remodeling.
α2β1	β1	Collagen (type I, II)	Primarily interact with collagen; Cell adhesion and migration in connective tissues.
α3β1	β1	Laminin, collagen	Bind laminin and collagen; important for epithelial cell adhesion and migration, wound healing.
α4β1	β1	Fibronectin, VCAM-1	Interact with fibronectin and VCAM-1; Immune cell trafficking, particularly in inflammation.
α5β1	β1	Fibronectin	Key in fibronectin binding; Cell migration and angiogenesis.
α6β1	β1	Laminin	Interact with laminin and epithelial cells; Basement membrane integrity.
α7β1	β1	Collagen	Interact with collagen; Muscle cell adhesion and migration
α8β1	β1	Laminin, Fibronectin	Bind fibronectin and laminin; Cell migration during embryonic development.
α9β1	β1	ECM	Important for immune cell migration and adhesion to fibronectin.
α10β1	β1	Collagen	Involved in collagen binding; Crucial for chondrocyte adhesion and cartilage formation.
α11β1	β1	Collagen	Functions similarly to α10β1; Collagen binding and cartilage remodeling.
αvβ1	β1	Fibronectin, laminin, gelatin, collagen	Interacts with fibronectin and collagen;
αLβ2	β2	ICAM-1, ICAM-2	Involved in cell migration, angiogenesis, immune response. On leukocytes; Immune cell adhesion and migration, important in inflammation.
αMβ2	β2	ICAM-1, C3b, Fibronectin	Involved in immune cell adhesion and migration, critical for phagocytosis and inflammation.
αXβ2	β2	ICAM-1, C3b	Participates in neutrophil adhesion to endothelial cells, inflammatory responses.
αDβ2	β2	ICAM-3	Involved in immune cell adhesion and migration, particularly in inflammation.
αvβ3	β3	Fibronectin, laminin, gelatin	Bind fibronectin, vitronectin, laminin; Important for vascular development, tumor cell migration, wound healing.
αIIbβ3	β3	Fibrinogen, collagen, fibronectin	Found on platelets; Critical for platelet aggregation and blood clotting (hemostasis).
α6β4	β4	Laminin	Involved in epithelial cell adhesion to basement membranes; Important for cell polarity and tissue architecture.
αvβ5	β5	Elastin, fibronectin	Bind vitronectin and fibronectin; Important for cell migration, angiogenesis, wound healing.
αvβ6	β6	Laminin	Bind laminin; Play a role in epithelial cell migration, inflammation, tissue remodeling.
α4β7	β7	VCAM-1, MAdCAM-1	Play a role in immune cell homing, particularly in the gut; Interacts with MAdCAM-1 in intestinal vasculature.
αEβ7	β7	E-cadherin	Involved in immune cell adhesion to E-cadherin in epithelial cells; Important for intestinal immunity.
αvβ8	β8	ECM, TGF-β	Involved in TGF-3 activation; Play a role in immune tolerance, inflammation, and tumor progression.

C3b: Complement component 3b; ECM: extracellular matrix; ICAM: intercellular cell adhesion molecule; MAdCAM-1: mucosal addressin cell adhesion molecule-1; TGF-β: transforming growth factor-β; VCAM-1: vascular cell adhesion molecule-1.

The conformational states of integrins and their bidirectional signaling are central to their core functions, enabling dynamic switching between inactive and active conformations. In the inactive/bent conformation, the α and β subunits remain closely associated, with the extracellular domains folded, leading to a low-affinity state for ligand binding (Pang et al., 2023b). Simultaneously, the transmembrane and cytoplasmic domains form a compact complex that prevents interactions with intracellular adaptors such as talin and kindlin (Luo et al., 2004; Springer and Dustin, 2012). Conversely, upon inside-out activation, typically triggered by the binding of talin/kindlin to the β-subunit cytoplasmic domain, integrins transition into the active/extended conformation, increasing their affinity for ECM ligands (Calderwood, 2004; Shattil et al., 2010). This extracellular engagement further induces outside-in signaling, promoting the recruitment of FAK, Src, and other downstream effectors, which collaboratively regulate cytoskeletal reorganization, cell survival, and transcriptional changes (Mitra and Schlaepfer, 2006; Piccinini and Midwood, 2010). By switching between these distinct conformational states, integrins facilitate rapid adaptation of cellular behavior in response to extracellular cues—an ability particularly critical within the CNS. In the CNS, the dynamic remodeling of synapses, glial processes, and neurovascular interfaces forms the basis for maintaining normal physiological function and responding to pathological challenges (Humphries et al., 2006). This dynamic signaling mechanism not only underscores the pivotal role of integrins in cell-ECM interactions but also highlights their fundamental importance in both neuronal health and disease.

Advancements in structural biology and artificial intelligence, particularly cryo-electron microscopy and AlphaFold technology (Hanein and Volkmann, 2018; Campbell et al., 2020; Adair et al., 2023; Abramson et al., 2024), have provided unprecedented insights into the precise conformational transitions of integrins from a low-affinity to a high-affinity state. These discoveries offer a structural basis for the rational design of integrin-targeted therapeutics, such as small-molecule allosteric modulators, which can selectively stabilize specific integrin conformations without completely abolishing integrin function (Slack et al., 2022; Roy et al., 2023; Mirabello et al., 2024). Furthermore, the interplay between integrins, growth factor receptors, and other membrane proteins suggests that integrins may serve as central nodes in neuroinflammation, angiogenesis, and synaptic plasticity-associated signaling networks (Hynes, 2002; Avraamides et al., 2008; Zhang and Wang, 2012; Biose et al., 2023). Understanding the interactions between specific integrin heterodimers and distinct CNS cell types remains a major research focus, as deciphering these interactions is crucial for identifying integrin subunits or complexes that can be therapeutically targeted in a disease-specific manner while preserving normal CNS function. In the subsequent sections, this review will delve into the roles of integrins in the healthy brain, neurodevelopment, and major neurological diseases, illustrating how their structural characteristics translate into functional outcomes.

### Distribution and role of integrins in the brain

**Figure 2:**
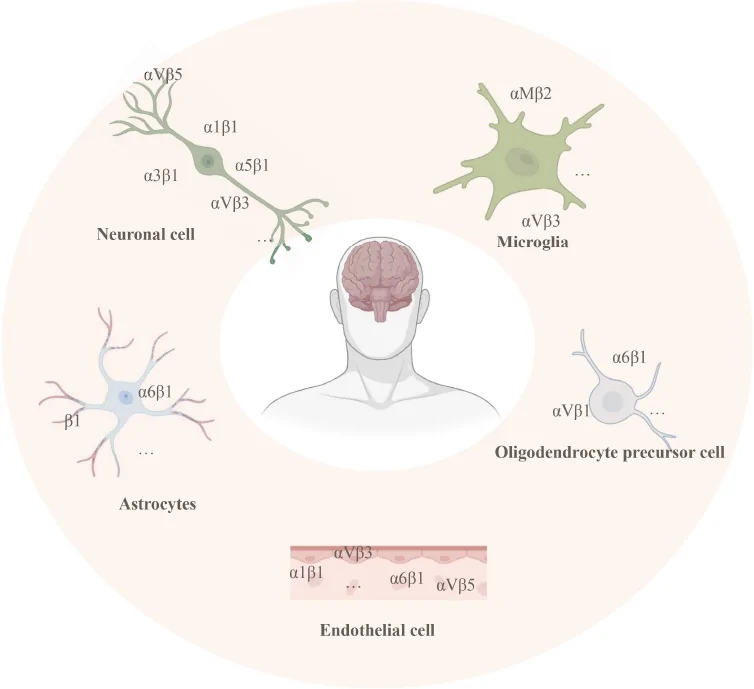
Distribution of integrins in the brain. Each cell type exhibits a unique integrin expression profile, with these integrins playing crucial roles in cell adhesion, migration, synaptic plasticity, and vascular integrity.

A growing body of evidence suggests that integrins play an essential role in the adult CNS, extending far beyond their well-established functions in peripheral tissues (Wu and Reddy, 2012; Lilja and Ivaska, 2018; Cui et al., 2024a). These transmembrane receptors are broadly expressed across major brain cell types, including neurons, astrocytes, microglia, oligodendrocytes, and vascular endothelial cells, where they orchestrate numerous processes critical for homeostasis, synaptic function, and neurovascular integrity (**Figure 2** and **Additional Table 3**).

**Additional Table 3:** Distribution and role of integrins in the brain

Cell type	Integrin subtypes	Function/role in the brain
Neurons	α1β1, α3β1, α5β1, αVβ3	Interact with ECM components like laminin and fibronectin; Important for neuron morphology, migration, synaptic plasticity, and learning and memory; Abnormal integrin function can lead to cortical layer defects and disordered neuronal migration.
Astrocytes	β1 (e.g., α6β1)	Expressed in astrocyte end-feet around brain blood vessels, crucial for BBB integrity, and regulate fluid and ion homeostasis; Involved in scarring and tissue repair after CNS injury.
Microglia	αMβ2 (Mac-1), αVβ3	Key for damage detection, adhesion to ECM, and phagocytosis; Regulate neuroinflammation, cytokine release, and neuron survival; Disruption is associated with neurodegenerative diseases.
Oligodendrocyte precursor cells	αVβ1	Involved in migration along ECM tracks, myelination, and remyelination after demyelinating injury; Dysfunction linked to multiple sclerosis and impaired myelin repair.
Endothelial cells	α1β1, α6β1, αVβ3, αVβ5	Involved in BBB maintenance, angiogenesis, and vascular remodeling; Regulate endothelial cell shape, permeability, and blood flow, critical for neurovascular coupling.
Overall CNS	Various (e.g., α1β1, αVβ3, α6β1)	Integrins coordinate interactions between neurons, glial cells, and blood vessels, ensuring CNS homeostasis, synaptic function, neurovascular coupling, and protection from harmful substances; Their dysregulation is implicated in CNS diseases like stroke, Alzheimer's disease, Parkinson's disease, and multiple sclerosis.

BBB: Blood-brain barrier; CNS: central nervous system; ECM: extracellular matrix.

Neurons express a diverse repertoire of integrin subtypes, including α1β1, α3β1, α5β1, and αVβ3, enabling interactions with key ECM components such as laminin and fibronectin (Wu and Reddy, 2012). These interactions are essential for neuronal morphology and migration. For instance, integrin α3β1 binding to laminin regulates cytoskeletal dynamics, promoting neurite outgrowth and axon guidance (Dulabon et al., 2000; Wu and Reddy, 2012), while the loss of β1 integrins in developing neurons can lead to defective cortical layering and impaired neuronal migration (Belvindrah et al., 2007; Rashid and Olson, 2023). In the context of synapse formation and plasticity, α3β1, α5β1, and αVβ3 integrins contribute to the assembly and maintenance of excitatory synapses (Banerjee et al., 1997; Firmo Morais, 2019; Wiera et al., 2022; Brzdąk et al., 2023; Ruggeri, 2024), partly by clustering synaptic vesicles and organizing the postsynaptic density. Additionally, integrins modulate synaptic plasticity by regulating long-term potentiation and long-term depression, thereby influencing learning and memory processes (Chan et al., 2003, 2006; Wiera et al., 2022). Disruption of integrin-ECM interactions has been implicated in synaptic dysfunction and cognitive deficits in animal models, highlighting their critical role in neuronal network stability and cognitive function.

Glial cells, including astrocytes, microglia, and oligodendrocytes, express several integrin subunits that are crucial for CNS maintenance and injury responses (Lathia et al., 2008; von Bernhardi et al., 2016). Astrocytic endfeet ensheath cerebral vasculature, forming an essential component of the BBB. Integrin β1 on astrocytes mediates adhesion to basement membrane components, ensuring BBB stability and regulating fluid and ion homeostasis (Ye et al., 2018; Yang et al., 2023). Additionally, integrins in astrocytes regulate scar formation and tissue repair following CNS injury, with integrin α6β1 being implicated in astrocyte migration and glial scar organization (Schmidt et al., 1996; Milner and Campbell, 2002; Yan and Cui, 2023). As the resident immune cells of the CNS, microglia rely on integrins such as αMβ2 and αVβ3 to sense injury signals, adhere to the ECM, and phagocytose cellular debris. Activation of microglial integrins triggers the release of cytokines and growth factors, influencing neuroinflammatory responses and neuronal survival. Dysregulated integrin signaling in microglia has been implicated as a potential driver of pathological inflammation in neurodegenerative diseases (Finneran and Nash, 2019). OPCs express integrins such as αVβ1, facilitating their migration along specific ECM trajectories (Milner et al., 1996). These integrins are essential for proper CNS axonal myelination and influence the efficiency of remyelination following demyelinating injury. Disruptions in integrin signaling may contribute to demyelinating pathologies such as MS (Maciak et al., 2023).

Cerebral endothelial cells express a diverse array of integrins, including collagen- and laminin-binding receptors (e.g., α1β1, α6β1) as well as RGD-recognizing αV integrins (e.g., αVβ3, αVβ5) (Halder et al., 2018; Hunter et al., 2022; Kuwar et al., 2023; Chen et al., 2024). These integrins serve critical functions in BBB maintenance, where endothelial cells form tight junctions reinforced by ECM-integrin interactions, ensuring selective permeability and protection against pathogens and toxins. Disruption of αVβ3 or β1 integrins has been shown to increase barrier permeability, facilitating edema and the influx of inflammatory mediators (Li et al., 2007; Alhadidi et al., 2024). In the context of angiogenesis and vascular remodeling, αVβ3 and αVβ5 integrins play pivotal roles in new blood vessel formation, particularly under hypoxic or injury conditions, where they coordinate with VEGF signaling to guide neovascularization and maintain proper vascular caliber in the adult brain (Kuwar et al., 2023; Chen et al., 2024). Moreover, integrin-mediated basement membrane adhesion influences endothelial cell morphology and mechanotransduction, thereby regulating blood flow dynamics. Proper neurovascular coupling, which ensures that local blood supply is matched to neuronal activity, is partially dependent on the stability and function of endothelial integrins (Aman and Margadant, 2023).

The coordinated expression of integrins across neurons, glial cells, and the cerebrovascular system is essential for maintaining overall CNS homeostasis. By anchoring neurons to specific ECM components, integrins contribute to synaptic stabilization, supporting efficient neurotransmission and plasticity (Lilja and Ivaska, 2018). Astrocytes and endothelial cells rely on integrins to establish a continuous, selectively permeable barrier, which shields the brain from potentially harmful substances while facilitating nutrient transport (Abbott, 2002; Desai et al., 2009; Manu et al., 2023). Furthermore, integrins play a crucial role in ensuring that metabolically active brain regions receive adequate blood supply, thereby coupling neuronal metabolism with vascular responses (Girouard and Iadecola, 2006; McCarty, 2009; Tanigami et al., 2012). In glial cells, integrins regulate astrocyte and microglial activation, migration, and neuroprotective functions, influencing scar formation, debris clearance, and trophic support for injured neurons (Yim et al., 2022; Milicevic et al., 2023; Wu et al., 2023b). Disruptions in these integrin-driven processes—whether due to genetic mutations, environmental insults, or disease-related factors—can compromise neuronal survival, BBB integrity, and neuroinflammatory balance. As a result, alterations in integrin expression or activity are frequently implicated in a wide range of CNS pathologies, including stroke, AD, PD, and MS.

### Integrin-mediated signaling pathways

#### Integrin-mediated “outside-in” and “inside-out” signaling

As transmembrane signaling hubs, integrins facilitate bidirectional information transfer—transducing signals from the extracellular environment into the cell (“outside-in” signaling) and conveying intracellular cues to the cell surface (“inside-out” signaling) (Kondo et al., 2022; Khatlani et al., 2023; Li et al., 2024a). The dynamic interplay between these processes enables integrins to rapidly modulate their ligand-binding affinity and influence multiple downstream events. In neurons, glial cells, and vascular cells within the CNS, integrin-mediated signaling is crucial for the regulation of cell adhesion, migration, differentiation, and tissue remodeling (Amatruda et al., 2022; Ikeshima-Kataoka et al., 2022; Halder et al., 2023; Morimoto et al., 2024; Yu et al., 2024). These signaling cascades are coordinated through the clustering of integrins at focal adhesion complexes, where integrins interact with cytoplasmic proteins such as FAK, ILK, talin, Kindlin, Paxillin, Vinculin, α-actinin, and other adaptor molecules (Burridge and Guilluy, 2016; Sun et al., 2019).

*Integrin-mediated “inside-out” signaling*

Inside-out signaling represents a complex intracellular mechanism through which intracellular cues trigger conformational changes in integrins, thereby enhancing their affinity for ECM ligands (Cappenberg et al., 2022; Wen et al., 2022; Li et al., 2024b). Upon stimulation by G protein-coupled receptor activation, cytokine receptor signaling, or localized second messengers, key cytoplasmic proteins such as talin and Kindlin bind to the β-subunit cytoplasmic tail of integrins (Moser et al., 2008; Siegel et al., 2022; Bojti et al., 2023; Wu et al., 2025). This interaction disrupts the salt bridge between the α- and β-subunit transmembrane/cytoplasmic domains, inducing a conformational switch that extends the extracellular domain of integrins, transitioning them into a high-affinity state for ligand binding (Shattil et al., 2010). Talin, a critical cytoskeletal adaptor, undergoes mechanically induced unfolding, exposing cryptic binding sites necessary for actin linkage and integrin activation (Sun et al., 2019). Kindlin, by binding to the membrane-proximal region of the β-subunit cytoplasmic tail, cooperates with talin to achieve full integrin activation (Moser et al., 2008). Notably, dysregulated Kindlin expression has been linked to axon guidance defects and abnormal glial responses in various CNS pathologies. In the CNS, inside-out integrin activation plays a crucial physiological role in synapse formation, synaptic plasticity, and cell motility. For instance, microglia dynamically adhere to or detach from sites of injury, a process that is partially dependent on the temporal regulation of integrin affinity (Milner and Campbell, 2002).

*Integrin-mediated “outside-in” signaling*

Outside-in signaling occurs when integrins adopt a high-affinity conformation and bind to their extracellular ligands, such as laminin, collagen, and fibronectin, leading to receptor clustering and the subsequent phosphorylation and activation of FAK and Src family kinases (Mitra and Schlaepfer, 2006). This activation initiates a cascade involving multiple downstream effectors. Activated FAK associates with Src kinases, leading to the phosphorylation of scaffold proteins such as Paxillin and p130Cas, which regulate cytoskeletal organization and cell migration (Mitra and Schlaepfer, 2006). Integrin-ligand engagement also recruits phosphatidylin-ositol-3-kinase (PI3K), subsequently activating protein kinase B (Akt), which supports neuronal survival and promotes neurite outgrowth and branching (Wang et al., 2023b; Jin et al., 2024). Furthermore, outside-in signaling stimulates the rat sarcoma (Ras)/v-raf murine sarcoma viral oncogene homolog (Raf)/mitogen-activated extracellular signal-regulated kinase (MEK)/extracellular signal-regulated kinase (ERK) axis, modulating gene expression programs related to cell proliferation, differentiation, and repair (Parashar et al., 2022; Koukourakis et al., 2023). Rho family GTPases (RhoA, Rac1, Cdc42) coordinate actin polymerization and focal adhesion turnover, influencing cell polarity, morphology, and motility (Neumann and Prekeris, 2023; Bailly et al., 2024; Morstein et al., 2024). Additionally, ILK interacts with Pinch and Parvin to form the ILK/PINCH/Parvin complex, a structural hub that integrates signals regulating actin cytoskeleton organization, proliferation, and neuronal migration (Legate et al., 2009; Ain and Firdaus, 2022; Mierke et al., 2022; Vaynberg and Qin, 2022). Through these interconnected pathways, integrins govern key processes in the CNS, including glial scar formation following injury, BBB stability, and synaptic connectivity during plasticity. Integrins function not only as chemical signal transducers but also as mechanosensors, converting mechanical forces at the cell surface into intracellular signaling cascades (Lei et al., 2023; Liu et al., 2024b). During CNS development, integrins play a crucial role by modulating focal adhesion dynamics and traction forces, thereby shaping neuronal migration pathways and the alignment of glial scaffolds, which are essential for tissue morphogenesis (Ridley, 2015). Following injury, such as stroke or traumatic lesions, integrin-mediated mechanosensing influences glial scar formation and vascular remodeling by regulating fibroblast and astrocyte proliferation and migration (Davis et al., 2023; Wen et al., 2023). Furthermore, increasing evidence suggests that integrin-dependent mechanotransduction modulates key transcription factors such as Yes-associated protein (YAP)/transcriptional coactivator with a PDZ-binding domain (TAZ) and β-catenin, thereby affecting neural stem cell fate, synaptic remodeling, and angiogenesis (Dupont et al., 2011; Elosegui-Artola et al., 2017).

In summary, integrins serve as central integrators of chemical and mechanical cues in the CNS, orchestrating a wide range of essential cellular processes. The interplay between inside-out and outside-in signaling endows cells with a high degree of adaptability, enabling them to rapidly respond to microenvironmental changes, which is crucial for maintaining CNS homeostasis and mounting effective responses to injury or disease (**Additional Table 4**; Parsons et al., 2010). Consequently, dysfunction in any component of these signaling cascades can have profound pathological consequences, potentially disrupting neuronal function, glial responses, and vascular integrity. The molecular mechanisms of integrin-mediated signaling in microglia, astrocytes, and neurons include key pathways such as FAK, PI3K/Akt, and MAPK, as well as emerging pathways.

**Additional Table 4:** Integrin-mediated "outside-in" and "inside-out" signaling

Signal direction	Mechanism	Key components/signaling molecules	Function/role in CNS
Inside-out signaling	Activation of integrins through intracellular signaling	Talin, Kindlin, β-integrin tail	Increase integrin affinity for ECM ligands, regulates cell adhesion and migration; Essential for synapse formation, synaptic plasticity, and glial response; Disruption can lead to axon guidance defects and glial cell response abnormalities.
Outside-in signaling	Integrin clustering upon binding to ECM ligands triggers downstream signaling	FAK, Src kinases, PI3K, Akt, Ras/Raf/ MEK/ERK, Rho GTPases (RhoA, Rac1, Cdc42), ILK	FAK and Src activation leads to cytoskeletal rearrangement and cell migration; Integrin-ECM binding activates PI3K/Akt for neuronal survival, neurite growth, and synapse function; Regulate glial scar formation, BBB stability, and synaptic connections during neuroplasticity.
Mechanosensation and mechanotransduction	Converts mechanical force at the cell surface into intracellular signaling	ILK, YAP/TAZ, β-catenin	Integrin-mediated mechanosensing modulates neuronal migration, glial scaffold arrangement, and tissue morphogenesis; Affect glial scar formation, vascular remodeling, and synaptic remodeling after CNS injury;
Crosstalk between signaling pathways	Integration of both chemical and mechanical signals	Talin, Kindlin, FAK, ILK, YAP/TAZ, β-catenin, Rho GTPases	Disruption impacts stem cell fate, synaptic remodeling, and angiogenesis. The bidirectional signaling between inside-out and outside-in pathways enables the CNS cells to adapt rapidly to environmental changes, crucial for CNS homeostasis, response to injury, and disease recovery.

Akt: Protein kinase B; BBB: blood-brain barrier; CNS: central nervous system; ECM: extracellular matrix; ERK: extracellular signal-regulated kinase; FAK: focal adhesion kinase; ILK: integrin-linked kinase; MEK: mitogen-activated extracellular signal-regulated kinase; PI3K: phosphatidylinositol 3-kinase; Raf: Raf kinase; Ras: Rat sarcoma; TAZ: transcriptional coactivator with PDZ-binding motif; YAP: Yes-associated protein.

#### Detailed mechanisms of integrin-mediated signaling

*Integrin-mediated FAK signaling pathway*

FAK is a critical molecule in integrin-mediated signaling, serving as a central hub in regulating cellular processes such as adhesion, migration, and survival. Upon integrin engagement with ECM components, FAK is recruited to focal adhesion sites, where it undergoes phosphorylation at specific tyrosine residues, including Tyr397. This phosphorylation event facilitates the recruitment of other signaling molecules, such as Src family kinases, and promotes downstream signaling pathways that control cell survival, migration, and cytoskeletal remodeling. (1) Microglia: FAK is vital for regulating microglial activation and migration, especially during neuroinflammatory responses. Recent studies have demonstrated that inhibition of Src kinase activity, which forms a functional complex with FAK in microglia, reduces the release of pro-inflammatory cytokines such as TNF-α and interleukin-1β, key mediators of neuroinflammation in neurodegenerative diseases including AD and PD (Dhawan and Combs, 2012). In models of AD, FAK inhibition led to a reduction in microglial-mediated neurotoxicity, thereby attenuating neuronal loss (Han et al., 2014; Saleh et al., 2022). Furthermore, FAK modulates the phagocytosis of Aβ plaques, which is crucial for clearing debris and modulating neuroinflammation in AD (Rong et al., 2020). (2) Astrocytes: FAK plays an important role in maintaining the integrity of the BBB, a critical function in neurovascular homeostasis. Recent studies have shown that FAK activation in astrocytes enhances BBB permeability under pathological conditions, such as IS, and prevents excessive leakage of plasma proteins into the CNS (Jia et al., 2022). Additionally, FAK signaling in astrocytes has been linked to neuronal survival and tissue repair after injury, particularly following ischemic events. FAK activation post-stroke has been shown to promote neuronal recovery and reduce infarct size by activating pro-survival pathways (Chen et al., 2018). (3) Neurons: FAK signaling in neurons regulates synaptic plasticity and neuroprotection (Monje et al., 2012). FAK signaling enhances neuronal resilience under stress by activating survival pathways such as PI3K/Akt (Wu et al., 2024). Disruption of FAK signaling in AD models has been shown to impair synaptic function and accelerate neurodegeneration (Saleh et al., 2022).

*Integrin-mediated PI3K/Akt signaling pathway*

The PI3K/Akt signaling cascade is one of the most crucial pathways activated by integrins. Upon integrin binding, PI3K is recruited to the membrane, where it catalyzes the conversion of phosphatidylinositol-4,5-bisphosphate (PIP2) to phosphatidylinositol-3,4,5-trisphosphate (PIP3), leading to the activation of Akt (Liu et al., 2023). Akt plays a key role in regulating cellular survival, growth, and metabolism by modulating various targets, including mammalian target of rapamycin (mTOR), Bcl-2 antagonist of cell death (Bad), and glycogen synthase kinase 3β (GSK3β) (Saxton and Sabatini, 2017). (1) Microglia: PI3K/Akt signaling in microglia is pivotal in balancing the survival of microglia during neuroinflammation (Tian et al., 2022). Recent findings suggest that PI3K/Akt signaling promotes microglial survival and resolves inflammation during early stages of neurodegenerative diseases like PD and AD (Li et al., 2023b; Wright et al., 2024). However, in later stages, prolonged activation of this pathway exacerbates chronic inflammation, thereby contributing to neurodegeneration (Chu et al., 2021). Modulating PI3K/Akt activation in microglia could therefore be a therapeutic strategy to either reduce inflammation or enhance tissue repair, depending on the clinical stage of the disease. (2) Astrocytes: PI3K/Akt activation in astrocytes promotes the release of neurotrophic factors such as brain-derived neurotrophic factor (BDNF) and glial cell line-derived neurotrophic factor (GDNF), which support neuronal survival and synaptic remodeling (Palasz et al., 2023). In IS models, enhanced PI3K/Akt activation in astrocytes facilitates the repair of injured tissue by inhibiting apoptosis and astrocyte reactivity (Gu et al., 2022). Additionally, in MS, PI3K/Akt signaling has been implicated in astrogliosis and the formation of glial scars, processes that can influence tissue recovery and remyelination (Theophanous et al., 2024). (3) Neurons: PI3K/Akt signaling is essential for neuronal survival, particularly under stress conditions such as oxidative damage. PI3K/Akt activation has been shown to protect dopaminergic neurons in PD models from neurodegeneration induced by α-synuclein (SNCA) aggregation (Nakano et al., 2017; Huang et al., 2020). In models of AD, Akt inhibition leads to impaired memory formation and neuronal loss (Yi et al., 2018).

*Integrin-mediated MAPK signaling pathway*

The MAPK pathway, which includes ERK1/2, c-Jun N-terminal kinase (JNK), and p38 MAPK, is critical for regulating cellular responses to stress, inflammation, and injury (Kyriakis and Avruch, 2012; Darling and Cook, 2014; Wang et al., 2021). Depending on the type of insult, MAPK signaling can promote either neuroinflammation or neuroprotection (Sawe et al., 2008). (1) Microglia: ERK1/2 activation in microglia has been shown to promote the production of pro-inflammatory cytokines such as TNF-α and IL-1β, exacerbating neuroinflammation in neurodegenerative diseases like AD and MS (Biswas, 2023). In contrast, inhibition of ERK1/2 in microglia has been found to reduce inflammation and protect against neuronal loss in these models (Lu et al., 2007; Shi et al., 2015). JNK and p38 MAPK activation in microglia contributes to neuronal apoptosis and the exacerbation of neurodegenerative processes (Lee and Kim, 2017). Targeting MAPK signaling in microglia could thus provide a potential therapeutic strategy to control neuroinflammation. (2) Astrocytes: p38 MAPK signaling is involved in reactive gliosis and the formation of glial scars following CNS injury. Studies have shown that p38 MAPK activation promotes the upregulation of GFAP, a marker of astrocytic activation, which is crucial for tissue repair (Saha et al., 2020). However, excessive or prolonged activation of this pathway can inhibit remyelination and hinder recovery (Chung et al., 2015). In MS, p38 MAPK signaling contributes to chronic inflammation and demyelination, suggesting that p38 MAPK inhibitors could be beneficial for promoting remyelination and reducing neuroinflammation (Lee et al., 2000). (3) Neurons: ERK1/2 activation in neurons is essential for maintaining synaptic integrity and neuronal survival. A study has demonstrated that ERK1/2 activation supports the survival of dopaminergic neurons in PD models by maintaining synaptic function and plasticity (Mutti et al., 2022). Furthermore, excessive activation of p38 MAPK and JNK pathways in neurons leads to oxidative stress-induced apoptosis, particularly in models of AD and Huntington’s disease (Yadav et al., 2021; Hosseini et al., 2024). Understanding the role of MAPK signaling in neurons may help identify therapeutic targets for preventing neuronal death and promoting neuronal resilience in neurodegenerative diseases.

*Integrin-mediated additional signaling pathways*

Recent research has uncovered additional signaling pathways that may be involved in integrin-mediated signaling in CNS cells, including the Hippo-YAP and Rho GTPase pathways. The Hippo-YAP pathway plays a key role in regulating cell proliferation and tissue repair, particularly in astrocytes and neurons (Ouyang et al., 2020). Studies have shown that the activation of YAP in response to integrin signaling promotes neuronal survival and tissue regeneration following CNS injury (Ikeshima-Kataoka et al., 2022; Yan and Cui, 2023). Similarly, Rho GTPases such as RhoA, Rac1, and Cdc42 regulate actin cytoskeletal dynamics and cellular migration in response to integrin engagement (Sadok and Marshall, 2014; Liu et al., 2024a). Recent findings have shown that Rho GTPases modulate microglial migration and phagocytosis in response to inflammatory stimuli, making them potential therapeutic targets in neuroinflammatory diseases like MS and AD (Guiler et al., 2021; Ahn and Park, 2025; **Additional Table 5**).

**Additional Table 5:** Detailed mechanisms of integrin-mediated signaling

Pathway	Key molecules/mechanisms	Function in CNS
FAK signaling	FAK, Src kinases, Paxillin, cytoskeletal remodeling, cell migration, phagocytosis of Aβ plaques	Regulate microglial activation, migration, neuronal survival, neuroinflammation, tissue repair.
PI3K/Akt signaling	PI3K, Akt, mTOR, Bad, GSK3β, BDNF, GDNF, neuronal survival, tissue repair, synaptic remodeling	Support neuronal survival, tissue repair, inflammation resolution, modulates neurodegeneration.
MAPK signaling	ERK1/2, JNK, p38 MAPK, Cytokine production, inflammation, apoptosis, synaptic plasticity	Regulate neuroinflammation, neuroprotection, synaptic integrity, neuronal apoptosis.
Hippo-YAP signaling	YAP, Hippo pathway, cell proliferation, tissue repair, neuronal survival	Promote neuronal survival, tissue regeneration, especially after CNS injury.
Rho GTPase signaling	RhoA, Rac1, Cdc42, actin cytoskeletal dynamics, microglial migration, phagocytosis	Modulate microglial migration and phagocytosis, influences neuroinflammation and injury response.

Akt: Protein kinase B; Bad: Bcl-2 antagonist of cell death; BDNF: brain-derived neurotrophic factor; CNS: central nervous system; ERK: extracellular signal-regulated kinase; FAK: focal adhesion kinase; GDNF: glial cell line-derived neurotrophic factor; GSK33: glycogen synthase kinase 3β; JNK: c-Jun N-terminal kinase; MAPK: mitogen-activated protein kinase; mTOR: mammalian target of rapamycin; PI3K: phosphatidylinositol 3-kinase; YAP: Yes-associated protein.

#### Molecular crosstalk between integrins and other pathways in neurological diseases

*Molecular crosstalk between integrins and cytokines in neurological diseases*

Cytokines are a group of signaling molecules that play a crucial role in regulating immune responses, inflammation, and tissue repair (Fan et al., 2023; Leung et al., 2023). In neurological diseases, cytokine signaling is often dysregulated, leading to chronic inflammation and neuronal damage. Integrins are central players in the regulation of neuroinflammation, as they mediate the adhesion and migration of immune cells across the BBB and into the CNS, where they interact with various cytokines to modulate the inflammatory response (Adamu et al., 2024; Mallick et al., 2025). TNF-α is a potent pro-inflammatory cytokine that is upregulated in response to brain injury, infection, or neurodegenerative processes. In diseases such as MS and AD, TNF-α is known to contribute to neuroinflammation and neuronal damage. Integrins, particularly α4β1, are essential for the recruitment of immune cells to sites of inflammation. In MS, for example, the binding of TNF-α to its receptors on immune cells can upregulate integrin expression, promoting the infiltration of T cells and macrophages into the CNS, where they exacerbate inflammation and damage to myelin (Severa et al., 2015). A study has shown that blocking α4β1 integrins or TNF-α signaling can reduce immune cell infiltration and improve outcomes in MS models (Lopes Pinheiro et al., 2016). The therapeutic potential of targeting integrin-TNF-α cross-talk is evident in the development of natalizumab, a mAb that blocks α4 integrins. This drug has shown effectiveness in reducing relapse rates and progression in MS patients, emphasizing the importance of integrin-TNF-α interactions in the disease’s pathogenesis (Mellergård et al., 2010). IL-6 is another key cytokine involved in neuroinflammation, especially in the context of neurodegenerative diseases such as AD and PD. IL-6 interacts with glial cells, such as astrocytes and microglia, and activates pro-inflammatory pathways that exacerbate neuroinflammation (Erta et al., 2012; Sulimai and Lominadze, 2020). The interaction between IL-6 and integrins leads to the upregulation of cytokine production and the activation of immune cells in the CNS (Keasey et al., 2018). Research has shown that certain integrins and IL-6 are highly expressed in AD, suggesting that their inhibition may attenuate microglial activation and neuronal damage in AD mouse models (Cai et al., 2022). The dual inhibition of IL-6 and integrins thus represents a promising strategy to reduce neuroinflammation and neuronal injury. Beyond TNF-α and IL-6, other cytokines such as IL-1β, interferon-γ (IFN-γ), and monocyte chemoattractant protein-1 (MCP-1) are involved in the regulation of neuroinflammation (Perrin et al., 2005; Eigenbrod et al., 2013). The crosstalk between these cytokines and integrins contributes to the recruitment of immune cells to the brain and exacerbates neurodegenerative processes.

*Molecular crosstalk between integrins and growth factors in neurological diseases*

Growth factors such as BDNF and GDNF are critical regulators of neuronal survival, differentiation, and synaptic plasticity (Nicoletti et al., 2022; Pisani et al., 2023). Integrins interact with these growth factors, enhancing neuronal survival and regeneration, which is particularly important in neurodegenerative diseases where tissue repair and neurogenesis are impaired (Wu and Reddy, 2012). BDNF is a neurotrophic factor that promotes neuronal survival and synaptic plasticity, and it is crucial for the maintenance of cognitive functions. Integrins, particularly α5β1, play a significant role in the release of BDNF in response to ECM signaling (Wu et al., 2018). BDNF binds to its receptor TrkB on neurons, activating downstream signaling pathways such as PI3K/Akt and MAPK/ERK, which protect neurons from apoptosis and promote synaptic plasticity (Shamsnia et al., 2025). Research has shown that integrin-mediated signaling can enhance BDNF release in the brain, thereby supporting neuronal survival in neurodegenerative diseases like PD and AD. For example, Tang et al. (2023a) demonstrated that integrin activation through fibronectin enhanced the neuroprotective effects of BDNF in a PD model. The activation of integrins by ECM proteins leads to the activation of FAK and PI3K/Akt signaling, which in turn may affect the secretion of BDNF (Wang et al., 2023b). This positive feedback loop is essential for maintaining neuronal health and plasticity, particularly in the context of neurodegenerative diseases. GDNF is another neurotrophic factor that plays a crucial role in the survival and regeneration of dopaminergic neurons, particularly in PD. Integrins, particularly β1 integrins, interact with GDNF receptors on dopaminergic neurons to promote neuroprotection and tissue repair (Chao et al., 2003). Integrin activation facilitated the binding of GDNF to its receptor Ret, activating intracellular survival pathways that protected dopaminergic neurons from degeneration (Chao et al., 2003; Cao et al., 2008). The ability of integrins to enhance the effects of neurotrophic factors like BDNF and GDNF highlights their potential for promoting neuronal regeneration and tissue repair. In neurodegenerative diseases, where neurogenesis and tissue repair are often impaired, targeting integrin signaling could enhance the regenerative capacity of neurons and glial cells (**Additional Table 6**).

**Additional Table 6:** Molecular crosstalk between integrins and other pathways in neurological diseases

Pathway	Key molecules/mechanisms	Function in CNS
Integrin-TNF-α crosstalk	α4β1 integrin, TNF-α, T cell and macrophage infiltration, neuroinflammation	Promote immune cell adhesion and migration, exacerbate neuroinflammation, and neuronal damage.
Integrin-IL-6 crosstalk	Cytokine, immune cells, neuroinflammation	Enhance cytokine production, promote microglial activation, worsen neurodegeneration.
Integrin-BDNF interaction	α5β1 integrin, BDNF, TrkB, PI3K/Akt, MAPK/ERK, neuronal survival, synaptic plasticity	Support neuronal survival, enhance synaptic plasticity, promote neuroprotection.
Integrin-GDNF interaction	β1 integrin, GDNF, Ret receptor, dopaminergic neurons, neuroprotection	Promote dopaminergic neuronal survival, tissue repair, and neuroprotection.

Akt: Protein kinase B; BDNF: brain-derived neurotrophic factor; CNS: central nervous system; ERK: extracellular signal-regulated kinase; GDNF: glial cell line-derived neurotrophic factor; IL-6: interleukin-6; MAPK: mitogen-activated protein kinase; PI3K: phosphatidylinositol 3-kinase; TNF-α: tumor necrosis factor α.

### Effects of integrins on neurodevelopment

Neurodevelopment is a highly coordinated process, encompassing neural cell proliferation, migration, differentiation, and the formation and refinement of synaptic connections (Marzola et al., 2023). Integrins have increasingly been recognized as key regulators of these processes, functioning both as structural anchors and signaling conduits that bridge the ECM with the intracellular cytoskeleton and downstream signaling pathways (Barros et al., 2011). During embryonic and postnatal development, integrins orchestrate fundamental cellular events, including radial migration, axon guidance, synaptogenesis, and vasculogenesis in the developing brain (Chen et al., 2022; Masuda et al., 2022; Johnson et al., 2023).

Radial migration of cortical neurons is a fundamental process in establishing the laminar structure of the cerebral cortex, requiring precise interactions between migrating neurons and radial glial cells (Marín and Rubenstein, 2003). Integrins expressed on neuronal growth cones, particularly integrin α3β1, facilitate neuron-glia adhesion by interacting with laminin and other ECM molecules along the radial glial scaffold (Dulabon et al., 2000). Studies have shown that mice lacking α3 integrin exhibit cortical layering defects, underscoring the critical role of α3β1-mediated signaling in guiding neurons to their correct laminar position (Schmid and Anton, 2003). Further investigations suggest that disruption of α3β1 function can lead to neurodevelopmental abnormalities (Primak et al., 2024; Saito-Diaz et al., 2024), such as lissencephaly-like phenotypes and impaired cortical circuit formation. Axon guidance and growth cone navigation are pivotal processes in neurodevelopment, wherein growth cones at the tips of developing axons interpret chemical and mechanical cues from the ECM to navigate toward their synaptic targets (Tessier-Lavigne and Goodman, 1996). Integrins play an essential role in this process, acting in coordination with guidance cues (e.g., netrins, semaphorins) and receptors (e.g., Robo, Eph) to finely tune axonal trajectories. Notably, integrins α1β1 and α5β1, which recognize collagen, fibronectin, and laminin, promote axon extension and branching (Kuwar et al., 2020; Su et al., 2022; Heino and Siljamäki, 2023). Additionally, axon guidance defects observed in β1 integrin knockdown models confirm the indispensable role of integrin signaling in stabilizing growth cones and ensuring accurate axonal navigation (Feltri et al., 2002; Lei et al., 2012). Synaptogenesis and synaptic maturation are essential for establishing functional neuronal connections. Following the initial contact between presynaptic and postsynaptic components, integrins—particularly those containing the β1 subunit—play a crucial role in synapse organization and stabilization (Wiera et al., 2022; Pang et al., 2023a). As a key regulator of synaptic stability, plasticity, and morphology, β1 integrin deletion disrupts synaptic vesicle clustering and postsynaptic receptor assembly, leading to deficits in long-term potentiation and altered synaptic plasticity (Chavis and Westbrook, 2001; Chan et al., 2003). Moreover, β1 integrin dysfunction has been linked to learning and memory impairments, further highlighting its role in synaptic refinement (Huang et al., 2006; You et al., 2022; Zhao et al., 2024). Cerebrovascular development is a complex process involving interactions among endothelial cells, astrocytes, and neurons, with integrin signaling playing a central guidance role. Specifically, αV integrins (e.g., αVβ3, αVβ5, αVβ8) are essential for angiogenesis, vascular stabilization, and astrocytic endfeet interactions (Hashiguchi et al., 2022; Pan et al., 2022; Kuwar et al., 2023). The loss of αV integrins can lead to vascular malformations, increased hemorrhagic risk, and disrupted BBB assembly. A previous study has suggested that αVβ8 integrin also participates in the activation of latent TGF-β, a process crucial for vascular remodeling and pericyte recruitment during embryonic brain development (Hirota et al., 2011). In early neurodevelopment, integrin subunit deficiencies or mutations can disrupt neuronal migration, axonal targeting, synaptic connectivity, and neurovascular integrity, predisposing individuals to a range of neurodevelopmental disorders, including cortical malformations, intellectual disability, and autism spectrum disorder. Additionally, postnatal integrin signaling defects may impair synaptic pruning and myelination, further compromising neuronal circuit maturation and plasticity (Warren et al., 2012).

Emerging tools, such as clustered regularly interspaced short palindromic repeats (CRISPR)/CRISPR-associated protein 9 (CRISPR/Cas9)-mediated gene editing and high-resolution live imaging techniques, are accelerating our understanding of integrin function in neural progenitor cells and newly formed synapses (Hazafa et al., 2020; Kalamakis and Platt, 2023; Lefebvre et al., 2024). Ongoing research aims to dissect how integrins integrate mechanical stimuli (e.g., tension from radial glial fibers) with chemical signals (e.g., growth factors and chemokines) to coordinate complex developmental events. In this context, the interactions between integrins and growth factor receptors (e.g., Trk, VEGF receptors) as well as guidance molecules (e.g., Netrin, Slit) remain a promising area of investigation. Ultimately, elucidating these integrin-dependent mechanisms may open new therapeutic avenues for neurodevelopmental disorders arising from impaired neuronal migration, defective axon guidance, or disrupted synaptogenesis (**Additional Table 7**).

**Additional Table 7:** Effects of integrins on neurodevelopment

Process	Integrin subtypes	Function/role in neural development
Radial migration of cortical neurons	α3β1	Involved in neuron-glial cell adhesion; Critical for the proper migration of neurons during cortical layer formation; Disruption of α3β1 leads to cortical layering defects.
Axon guidance and growth cone navigation	α1β1, α5β1	Integrins interact with ECM components (collagen, fibronectin, laminin) to promote axon elongation and branching.
Synaptogenesis and synaptic maturation	β1	Play a key role in the formation and stabilization of synaptic connections; Knockout of β1 leads to impaired synaptic vesicle clustering, affecting synaptic plasticity and LTP
Vascular development and angiogenesis	αVβ3, αVβ5, αVβ8	Essential for vascular development, angiogenesis, and BBB maintenance; αVβ8 also activates latent TGF-β important for vascular remodeling and pericyte wrapping.
Neuronal migration and axon targeting	α6β1, αVβ1	Facilitate neuronal migration along specific ECM tracks and is required for axon guidance; Defect can lead to abnormal cortical development and axon pathfinding.
Synaptic plasticity and stability	β1	Critical for synapse stability, plasticity, and morphology; Loss of β1 impairs synaptic function and learning and memory.
Neurovascular integrity and function	αVβ3, αVβ5	Key for the interaction between endothelial cells and astrocytes, maintaining BBB stability and vascular remodeling; Loss of αV integrins can lead to vascular malformation and increased bleeding risk.
Impact of integrin dysregulation	Various (e.g., α3β1, β1, αV)	Mutations or deficiencies in integrin subunits can alter neuron migration, axon guidance, synapse formation, and neurovascular integrity, leading to neurodevelopmental disorders like intellectual disabilities and autism.

BBB: Blood-brain barrier; ECM: extracellular matrix; LTP: long-term potentiation; TGF-β: transforming growth factor β.

## Integrins and Neurological Diseases

### Role of the integrin family in stroke

Stroke is one of the most prevalent and devastating neurological disorders worldwide, exerting profound personal, societal, and economic burdens (Katan and Luft, 2018; GBD 2019 Stroke Collaborators, 2021). Stroke is broadly classified into IS, which accounts for approximately 85% of all cases, and HS, comprising the remaining 15%. Both subtypes are characterized by acute focal neurological deficits, yet their underlying pathophysiological mechanisms differ substantially (Hakimi and Garg, 2016; Lui et al., 2025). In IS, arterial occlusion leads to localized hypoperfusion and tissue ischemia, whereas HS results from extravasation of blood into the brain parenchyma or subarachnoid space, causing tissue damage, inflammation, and increased intracranial pressure (Donnan et al., 2008; Cordonnier et al., 2018). This section provides an in-depth analysis of the latest advances in understanding the role of integrins in the pathophysiology of IS, while also offering additional insights into HS.

#### Current research on the integrin family in ischemic stroke

*The role of the integrin family in the inflammatory response to ischemic stroke*

Integrins play a pivotal role in the inflammatory response following IS, where inflammation exerts both detrimental and reparative effects on stroke outcomes (Candelario-Jalil et al., 2022; Wang et al., 2023a). Integrins expressed on circulating leukocytes and endothelial cells are essential for mediating inflammatory cell adhesion, transendothelial migration, and subsequent tissue responses (Zhu et al., 2022).

Integrin β1 plays a crucial role in the inflammatory response following IS by regulating leukocyte adhesion, migration, and BBB permeability (Edwards et al., 2020; Janus-Bell et al., 2024; Scalise, 2024; Tang et al., 2024; Zhang et al., 2024b). Integrin α4β1 (also known as CD49d/CD29 or VLA-4) is primarily expressed on leukocytes, where its activation is associated with chemokine interactions (Ghandour et al., 2007). Integrin α5β1 modulates the BBB by interacting with fibronectin and regulating the expression of matrix metalloproteinase-9 (Milner et al., 2007). Elevated matrix metalloproteinase-9 activity disrupts tight junction proteins, exacerbating vascular permeability and edema (Lee et al., 2024a; Sunny et al., 2024). As demonstrated by Edwards et al. (2020), inhibiting integrin α5β1 significantly reduces infarct volume, edema, and neurological deficits following IS. Administration of ATN-161, an α5β1 integrin inhibitor, attenuated IgG extravasation via claudin-5, collagen IV, and CXCL12, while also lowering matrix metalloproteinase-9 transcription levels, suggesting that α5β1 inhibition mitigates BBB disruption and post-IS inflammation. Integrin α9β1 is highly expressed on activated neutrophils, contributing to stable adhesion (Barbhuyan et al., 2024; Yuan et al., 2024a). Dhanesha et al. (2020) reported that targeting myeloid-specific α9β1 integrin improves both short- and long-term functional outcomes in IS models with comorbidities by limiting cerebral thrombosis and inflammation.

Dysfunction of β2 integrins has been associated with improved functional outcomes and reduced expression of pro-inflammatory cytokines in the brain. Integrin αLβ2 (lymphocyte function-associated antigen-1) and αMβ2 bind to intercellular adhesion molecule-1 on activated endothelial cells, facilitating firm leukocyte adhesion and extravasation (Ou et al., 2021). The role of αLβ2 integrin in IS involves leukocyte adhesion, migration, and subsequent inflammatory responses. Expressed on all leukocytes, particularly T lymphocytes (Hammond et al., 2014; Walling and Kim, 2018), αLβ2 mediates leukocyte adhesion to the vascular wall via intercellular adhesion molecule-1 interactions (Park et al., 2010). Moreover, αLβ2 integrin activation and conformational shifts to a high-affinity state are linked to G protein-coupled receptor and Rap-1 activation, leading to leukocyte rolling, adhesion, and exacerbation of inflammatory injury (Ghandour et al., 2007). Additionally, Harjunpää et al. (2024) reported that αMβ2 integrin contributes to post-IS leukocyte upregulation, and its inhibition reduces neutrophil infiltration and infarct volume. Tuttolomondo et al. (2009) demonstrated that ATP depletion via CD39-mediated mechanisms attenuates P2X7 receptor activation, thereby reducing baseline leukocyte αMβ2 integrin expression. These findings suggest that blocking αMβ2 integrin may reverse the post-ischemic inflammatory phenotype observed in Cd39-deficient mice.

**Figure 3:**
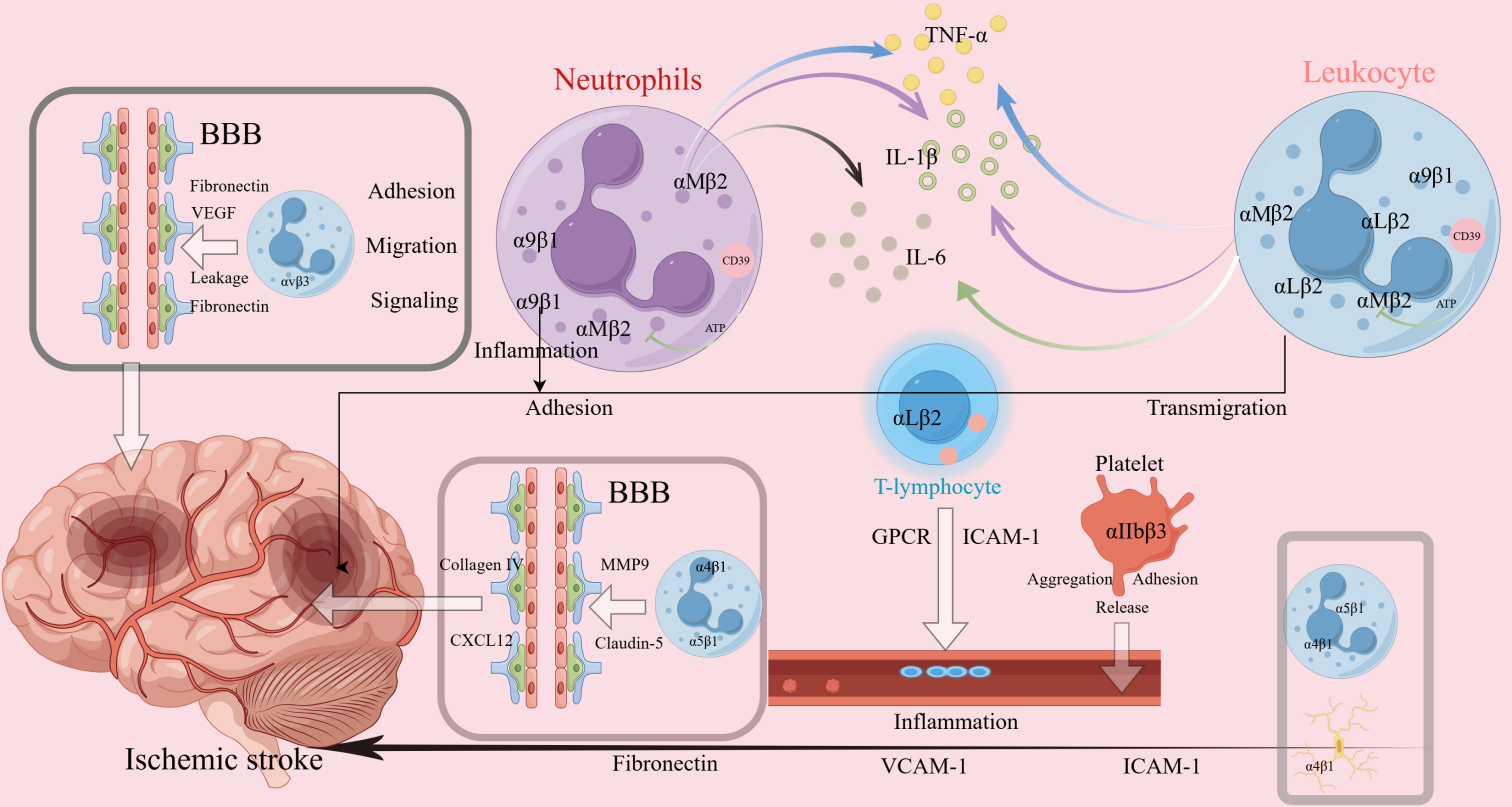
Members of the integrin family regulate the process of IS by participating in inflammatory processes. The expression of integrins on leukocytes and endothelial cells is crucial for the adhesion, migration of inflammatory cells, and tissue responses. Notably, integrins β1, α4β1, α5β1, α9β1, αLβ2, and αMβ2 are involved in regulating BBB permeability and inflammatory processes. Furthermore, integrin β3, particularly αIIbβ3 on platelets, plays an essential role in hemostasis and resolution of inflammation. Modulating the activity of these integrins may help control inflammatory responses and mitigate pathological damage associated with ischemic stroke. Created with Figdraw 2.0 (export code: TWPSA6e554). BBB: Blood–brain barrier; CXCL12: chemokine C–X–C motif ligand 12; GPCR: G protein-coupled receptors; ICAM-1: intercellular cell adhesion molecule-1; IL: interleukin; IS: ischemic stroke; MMP9: matrix metalloproteinase-9; TNF-α: tumor necrosis factor-α; VCAM-1: vascular cell adhesion molecule 1; VEGF: vascular endothelial growth factor.

Integrin β3 plays a significant role in the inflammatory processes of IS. Integrin αIIbβ3 (also known as GPIIb/IIIa) is the most abundantly expressed integrin on the platelet membrane, where it serves a pivotal function in hemostasis, mediating platelet adhesion, aggregation, and secretion (Montecino-Garrido et al., 2024; Rodriguez Moore et al., 2024). Senchenkova et al. (2019) reported that Annexin A1 modulates αIIbβ3 activation, shifting platelet phenotypes from a pro-pathological state to a regulatory state during ischemia-reperfusion injury. This transition reduces platelet aggregation and thrombosis, while promoting inflammation resolution and neuroprotection. Integrin αVβ3 plays a crucial role in various biological processes, including cell adhesion, migration, and signal transduction. Bi and Yi (2014) demonstrated that αVβ3 integrin inhibition blocks VEGF-induced BBB leakage, attenuates inflammatory responses, and reduces fibrinogen deposition. These findings suggest that modulating integrin activity may serve as a therapeutic strategy to regulate inflammation and mitigate IS-related pathology (**Figure 3** and **Additional Table 8**).

**Additional Table 8:** The role of integrins in various neurological disorders

Disease	Integrin	Mechanisms
Ischemic stroke	α4β1	Integrin α4β1 is predominantly expressed on leukocytes, and its activation is associated with interactions with chemokines.
	α5β1	α5β1 regulates the BBB by interacting with fibronectin and controlling the expression of MMP-9.
		Inhibition of integrin α5β1 significantly reduces infarct volume, edema, and neurological deficits following ischemic stroke.
		The α5β1 integrin inhibitor ATN-161 reduces IgG extravasation through claudin-5, collagen IV, and CXCL12, while also lowering the transcription levels of MMP-9.
		Inhibitors of integrin α5β1 suppress apoptosis by modulating the levels of p-FAK and p-AKT, thereby improving ischemic stroke.
		Overexpression of integrin α5β1 is associated with the promotion of angiogenesis and the integrity of the BBB.
		The administration of domain V following IS increases VEGF levels through a mechanism involving α5β1 integrin on brain endothelial cells; subsequently, the neuroprotective and angiogenic effects of domain V are mediated through VEGFR.
	α9β1	Integrin α9β1 is highly expressed on activated neutrophils, promoting stable adhesion. Targeting myeloid-specific α9β1 integrins improves both short-term and long-term functional outcomes in ischemic stroke models by limiting cerebral thrombosis and inflammation.
	αLβ2	αLβ2 is expressed on all leukocytes, particularly T lymphocytes, and mediates leukocyte adhesion to the vascular wall through interactions with ICAM-1. Additionally, the activation and conformational shift of αLβ2 integrin to a high-affinity state are associated with GPCR and Rap-1 activation, leading to leukocyte rolling, adhesion, and exacerbated inflammatory injury.
	αMβ2	ATP depletion via CD39-mediated mechanisms attenuates P2X7 receptor activation, thereby reducing baseline leukocyte αMβ2 integrin expression.
	αIIbβ3	Annexin A1 modulates αIIbβ3 activation, shifting platelet phenotypes from a pro-pathological state to a regulatory state during ischemia/ reperfusion injury.
	αVβ3	After ischemia, the expression of αVβ3 integrin is upregulated, playing an active role in the recovery process after ischemia by promoting vascular regeneration and neural repair
		αVβ3 integrin inhibition blocks VEGF-induced BBB leakage, attenuates inflammatory responses, and reduces fibrinogen deposition.
	αM	ITGAM significantly influences neuronal apoptosis, interference with ITGAM inhibits cellular apoptosis, while its overexpression promotes it.
	β1	Upregulating integrin β1 and modulating the β1/PI3K/Akt signaling pathway can ameliorate neurological dysfunction, mitigate oxidative stress injury, and reduce neuronal apoptosis in rats subjected to middle cerebral artery occlusion/reperfusion.
		Postischemic angiogenesis can facilitate neuronal remodeling and is believed to contribute to functional recovery. This is attributed to the role of newly formed vessels postischemia in guiding sprouting axons through the signaling of VEGF and laminin/β1-integrin.
		β1 integrin receptors can directly contribute to BBB dysfunction.
Hemorrhagic stroke	αIIbβ3	Modulating αIIbβ3 activity may help control thrombus expansion and prevent rebleeding.
	β2	In experimental hemorrhagic stroke models, pharmacological or genetic blockade of β2 integrins has demonstrated neuroprotective potential, reducing neuroinflammation and tissue damage.
	αV	Integrin αV plays a role in the inflammatory response and tissue repair following ICH. Integrin αV may support vascular integrity and mitigate BBB disruption in hemorrhagic lesions.
Alzheimer's disease	αMβ2 and αVβ3	αMβ2 and αVβ3 integrins promote the adhesion, migration, and uptake of Aβ aggregates; αMβ2 plays an important role in guiding microglial migration and facilitating β3 plaque phagocytosis.
	β1	β1 integrin signaling regulates APP trafficking and secretase activity. Aβ oligomers can interact directly or indirectly with β1 integrins at synapses, leading to morphological changes, dendritic spine retraction, and impaired LTP Stabilizing or upregulating β1 integrin expression has shown promising results in rescuing synaptic deficits. Enhancing β1 integrin signaling restores dendritic spine density, improves synaptic plasticity, and enhances cognitive performance.
	αVβ5 and αVβ8	αVβ5 and αVβ8 regulate the activation of latent TGF-β, modulating astrocytic and local microenvironmental states toward either pro-inflammatory or anti-inflammatory conditions.
Parkinson's disease	β2 (e.g., αMβ2, αXβ2)	High levels of β2 integrins (such as αMβ2, αXβ2) are expressed, and these integrins promote microglial adhesion and migration towards degenerating neurons or α-synuclein inclusions, where they may exacerbate oxidative stress and release pro-inflammatory cytokines.
	αVβ3	αVβ3 integrin interacts with RGD-containing ECM proteins (such as osteopontin, and vitronectin) and growth factors, activating downstream key pathways that promote neuronal survival and plasticity, including FAK and PI3K/Akt signaling pathways.
Multiple sclerosis	α4 and β1	β1 integrins on OPCs are essential for remyelination, regulating OPC migration, proliferation, and differentiation within demyelinated lesions. The α4-VCAM-1 interaction enables T cells to roll and firmly adhere to activated CNS endothelial cells; disruption of α4 function significantly reduces T cell infiltration and CNS lesion formation, thereby alleviating demyelination and inflammation.
	αMβ2 and αXβ2	β2 integrins, including αMβ2 and αXβ2, are expressed on activated microglia and infiltrating macrophages, where they regulate the phagocytosis of myelin debris.
	αLβ2	αLβ2 plays a crucial role in T cell extravasation, facilitating T lymphocyte migration across the BBB through interactions with ICAM-1; targeting αLβ2 may selectively reduce T cell infiltration.
Traumatic brain injury	α3β1	TIMP2 reduces neurodeficits and BBB leakage in TBI mice by interacting with α3β1 integrin on endothelial cells, inhibiting Src-dependent VE-cadherin phosphorylation.
	β2	Blocking β2 integrin improves neurofunction in TBI mice, reducing BBB permeability, oxidative stress, and inflammatory mediator release, while enhancing cerebral perfusion.
	αM	Blocking fibrinogen- CD11b interaction decreases monocyte recruitment and inflammation/ROS pathway activation in microglia and macrophages post-TBI, alleviating oxidative damage and cortical loss.
	β1	Drebrin loss impairs scarring and causes excessive neurodegeneration, affecting β1-integrin transport.
Neurodevelopmental disorders	αX	Prenatal inflammation damages early CD11c-positive microglia, hindering myelination in neurodevelopmental disorders, with a lack of high-CD11c microglia observed in affected children.
	αVβ8	αVβ8 deficiency in the CNS prevents normal microglia maturation, resulting in unique neurodevelopmental syndromes characterized by oligodendrocyte maturation arrest, interneuron loss, and spastic motor dysfunction.

Akt: Protein kinase B; APP: amyloid precursor protein; BBB: blood-brain barrier; CNS: central nervous system; CXCL12: chemokine C-X-C motif ligand 12; ECM: extracellular matrix; FAK: focal adhesion kinase; GPCR: G protein-coupled receptors; ICAM-1: intercellular cell adhesion molecule-1; ICH: intracerebral hemorrhage; LTP: long-term potentiation; MMP- 9: matrix metalloproteinase-9; OPC: oligodendrocyte precursor cell; p-AKT: phosphorylated protein kinase B; p-FAK: phosphorylated focal adhesion kinase; PI3K: phosphatidylinositol 3-kinase; RGD: Arg-Gly-Asp; TBI: traumatic brain injury; TIMP2: tissue Inhibitor of metalloprotease-2; TGF-β: transforming growth factor β; VCAM-1: vascular cell adhesion molecule-1.

*Role of the integrin family in the apoptotic process in ischemic stroke*

**Figure 4:**
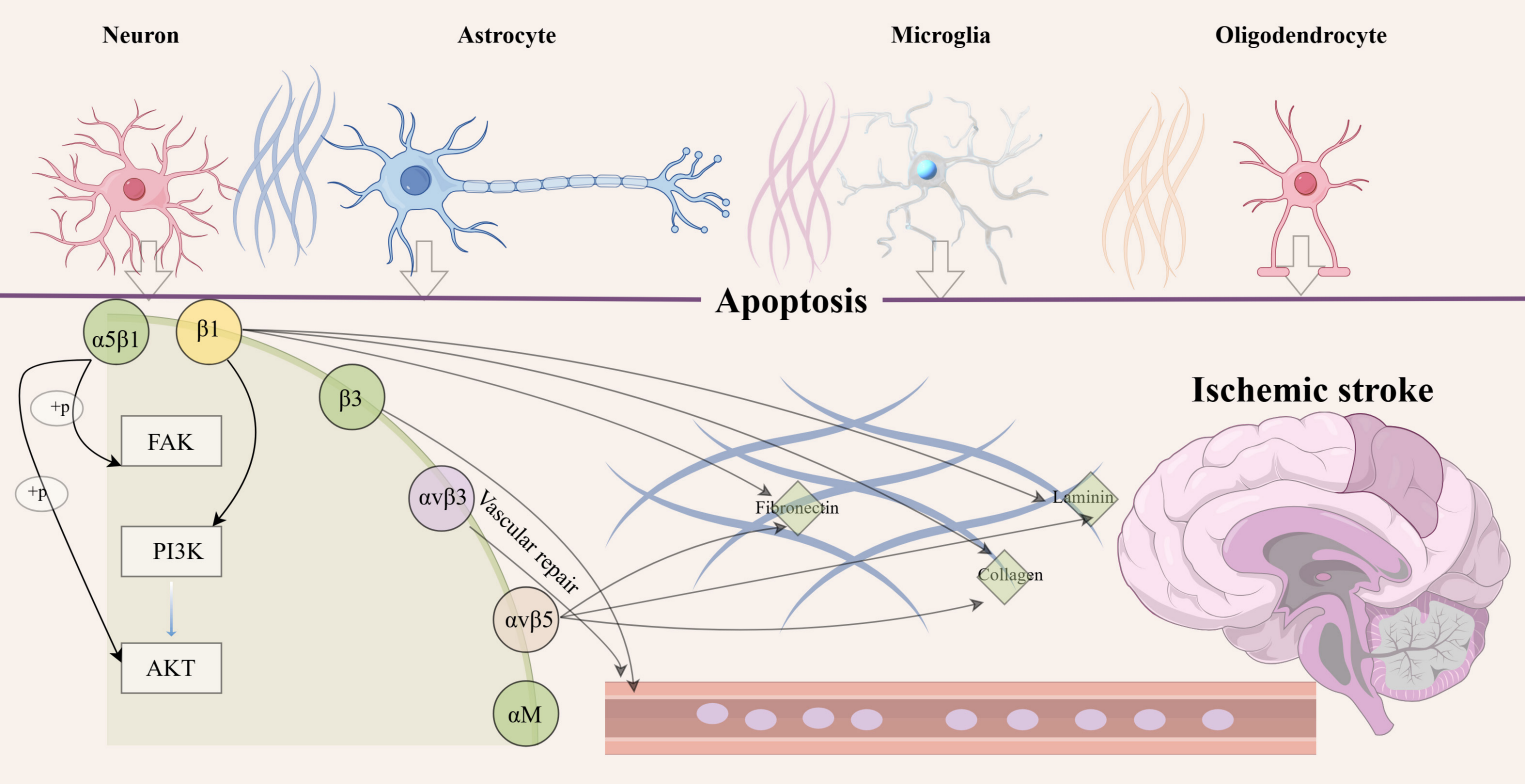
Members of the integrin family regulate the process of IS by participating in the apoptotic process. Interactions between integrins and the ECM activate survival signaling pathways such as FAK and PI3K/Akt, which inhibit apoptosis and promote cell survival. Under ischemic conditions, these pathways are impaired, leading to apoptosis. Modulation of integrins α5β1 and β1 affects p-FAK and p-AKT, thereby inhibiting apoptosis and facilitating vascular repair and neuroprotection. Expression of integrin β3 increases post-IS and is associated with vascular repair. Integrins play a crucial role in the pathophysiology of IS, and their activity modulation may offer novel therapeutic strategies. Created with Figdraw 2.0 (export code: IRIRA0e866). AKT: Protein kinase B; ECM: extracellular matrix; FAK: focal adhesion kinase; IS: ischemic stroke; p-AKT: phosphorylated protein kinase B; p-FAK: phosphorylated focal adhesion kinase; PI3K: phosphoinositide-3 kinase.

During the occurrence of IS, the altered dependence of neurons and glial cells on the ECM is a crucial trigger for initiating the apoptotic process. Integrins regulate cell adhesion, survival, and migration by binding to ECM components such as laminin, fibronectin, and collagen. Under ischemic conditions, integrin expression and signaling pathways are compromised, leading to reduced cell adhesion and the induction of anoikis. Integrins interact with the ECM to activate downstream survival signaling pathways, such as the FAK and PI3K/Akt signaling axes. Under normal conditions, these pathways can promote cell survival by inhibiting the activity of proapoptotic proteins such as Bax and Bad. However, during ischemic brain injury, the integrin-ECM interaction is disrupted, and the FAK and PI3K/Akt signaling pathways are affected, driving cells toward apoptosis (Mei et al., 2024). For example, Hou et al. (2024) reported that ITGAM significantly influences neuronal apoptosis, interference with ITGAM inhibits cellular apoptosis, while its overexpression promotes it. β1 integrin, one of the most common integrin subtypes, plays a significant role in neuronal adhesion and survival (Sekine et al., 2012). Yang et al. (2022) demonstrated that upregulating integrin β1 and modulating the β1/PI3K/Akt signaling pathway can ameliorate neurological dysfunction, mitigate oxidative stress injury, and reduce neuronal apoptosis in rats subjected to middle cerebral artery occlusion/reperfusion. In the regulation of apoptosis, integrin α5β1 can affect the levels of phosphorylated focal adhesion kinase (p-FAK) and phosphorylated protein kinase B (p-AKT). Upon activation of integrin α5β1, it can trigger the autophosphorylation of FAK, which activates PI3K, leading to the phosphorylation and activation of AKT (Pang et al., 2023b). Amruta and Bix (2021) reported that inhibitors of integrin α5β1 can ameliorate IS by suppressing apoptosis via the modulation of p-FAK and p-AKT levels. β3 integrin is crucial for platelet aggregation and vascular endothelial cell function. Bi and Yi (2014) have shown that the expression of β3 integrin increases after IS, which may be related to the repair of cerebral blood vessels and the formation of new blood vessels. The αvβ3 integrin heterodimer is involved primarily in angiogenesis. After ischemia, the expression of αvβ3 integrin is upregulated, playing an active role in the recovery process after ischemia by promoting vascular regeneration and neural repair. These findings indicate that integrins play a significant role in the apoptotic processes of IS, and by modulating the activity of integrins, the apoptotic processes of the organism can be regulated, thereby exerting a therapeutic or mitigating effect (**Figure 4** and **Additional Table 8**).

*Role of the integrin family in the oxidative stress response in ischemic stroke*

Brain tissue undergoes hypoxia and nutrient deprivation in the IS, leading to increased oxidative stress. Oxidative stress induces the accumulation of reactive oxygen species (ROS) within cells, which can damage cellular membranes, proteins, and DNA, ultimately resulting in cellular dysfunction and apoptosis. Integrins, through their interaction with the ECM, activate downstream survival signaling pathways, such as the FAK pathway (Kanchanawong and Calderwood, 2023). These signaling pathways can influence the expression of genes related to oxidative stress, thereby mitigating the damage to cells caused by oxidative stress. Additionally, integrins may exert their effects by regulating the expression of genes associated with oxidative stress.

**Figure 5:**
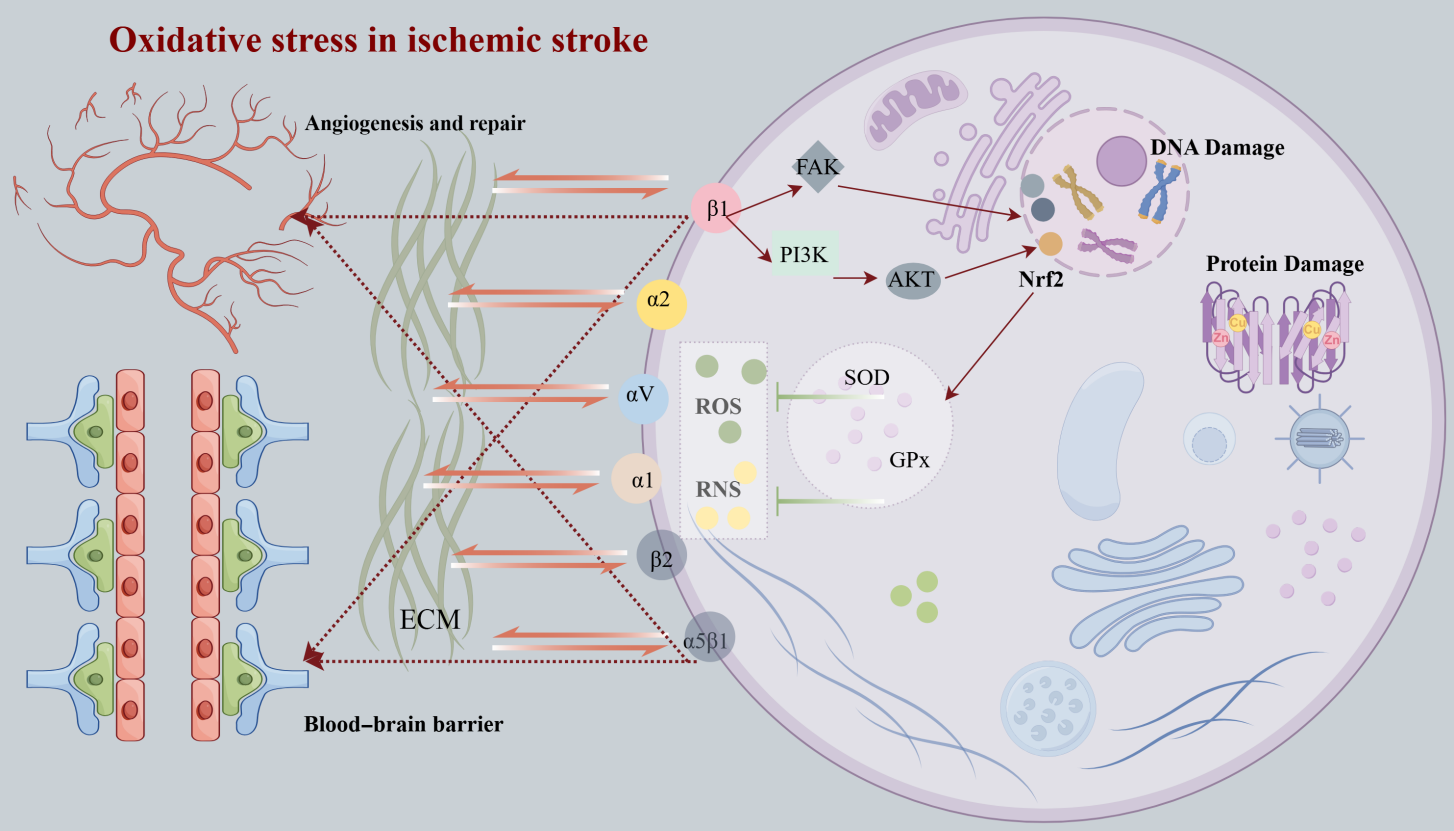
Members of the integrin family regulate the process of IS by participating in the oxidative stress response process. During IS, oxidative stress increases ROS, leading to cellular damage, dysfunction, and apoptosis. Integrins activate survival signaling pathways such as FAK and PI3K/Akt, modulating the expression of antioxidant genes to mitigate oxidative stress damage. Notably, integrins facilitate the nuclear translocation of Nrf2, enhancing antioxidant defenses. They may also alleviate brain injury by regulating cell death pathways. Antioxidants improve clinical outcomes by modulating integrin-associated signaling pathways, highlighting the potential of integrins in the treatment of IS. Created with Figdraw 2.0 (export code: TWWOUTe61a). AKT: Protein kinase B; ECM: extracellular matrix; FAK: focal adhesion kinase; GPx: glutathione peroxidase; IS: ischemic stroke; Nrf2: nuclear factor erythroid 2-related factor 2; PI3K: phosphoinositide-3 kinase; RNS: reactive nitrogen species; ROS: reactive oxygen species; SOD: superoxide dismutase.

Integrin family members influence oxidative stress through various mechanisms, thereby affecting the occurrence and progression of IS. Integrins can modulate signaling pathways associated with oxidative stress, such as the PI3K/Akt pathway. For example, Yang et al. (2022) reported that upregulating the Integrin-β1/PI3K/Akt signaling pathway can prevent oxidative stress, thereby alleviating IS. Another study has indicated that the overexpression of integrin α5β1 is associated with the promotion of angiogenesis and the integrity of the BBB (Wang et al., 2019). Furthermore, cell death induced by oxidative stress, such as necrosis or necroptosis (Uttara et al., 2009), may be regulated by integrin signaling pathways following the occurrence of IS. Integrins may mitigate brain injury by affecting cell death pathways. In clinical trials, antioxidants have been used to alleviate BBB disruption and improve clinical outcomes by inhibiting oxidation or the production of ROS (Lochhead et al., 2024). These antioxidants may exert their effects by modulating integrin-associated signaling pathways (**Figure 5** and **Additional Table 8**).

*Role of the integrin family in autophagy in ischemic stroke*

Integrin-mediated cell adhesion can influence the process of autophagy. The loss of attachment of cells to the ECM may induce autophagy, which, similar to its established cytoprotective role during nutrient starvation, can protect cells from detachment-induced cell death. Studies have shown that intracellular signals associated with integrins modulate autophagic responses and determine the balance between cell death and survival following the loss of cell-matrix adhesion (Zhao et al., 2022). Under hypoxic conditions, integrin signaling pathways can induce resistance to anoikis by regulating the expression of activating transcription factor 4 (ATF4) and autophagy-related genes (Pang et al., 2023b).

**Figure 6:**
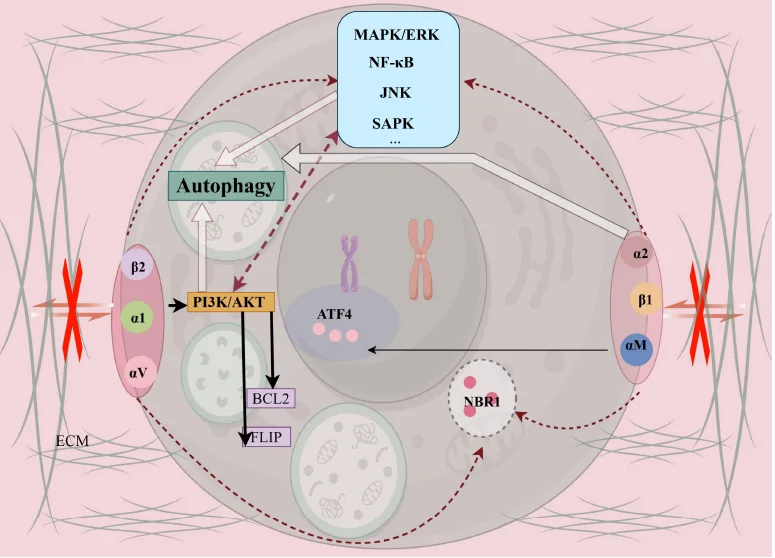
Members of the integrin family regulate the process of the IS by participating in the autophagy process. Integrin signaling modulates autophagy during cell detachment, affecting cell survival. Under hypoxic conditions, integrins regulate the expression of ATF4 and autophagy-related genes, resisting anoikis. In IS, integrins activate signaling pathways such as p53, Bcl-2, FLIP, MAPK/ERK, PI3K/AKT, JNK, SAPK, and NF-κB, controlling autophagy and cell fate. Post-stroke, OPN and Beclin1 increase, promoting the expression of integrin β1 and LC3, enhancing neural plasticity, and offering a novel therapeutic strategy for IS. Created with Figdraw 2.0 (export code: TIITY3dd17). AKT: Protein kinase B; ATF4: activating transcription factor 4; Bcl-2: B cell lymphoma-2; ECM: extracellular matrix; ERK: extracellular signal-regulated kinase; FLIP: FLICE inhibitory protein; IS: ischemic stroke; JNK: c-Jun N-terminal kinase; LC3: microtubule-associated protein 1 light chain 3; MAPK: mitogen-activated protein kinase; NBR1: neighbor of BRCA1 gene 1; NF-κB: nuclear factor kappa-B; OPN: osteopontin; PI3K: phosphoinositide-3 kinase; SAPK: stress-activated protein kinase.

In IS, the activation of integrin signaling pathways can induce the activation of p53; upregulate the expression of prosurvival molecules such as Bcl-2 and FLICE inhibitory protein (FLIP); and activate the MAPK/ERK pathway, PI3K/AKT pathway, JNK signaling pathway, and the stress-activated protein kinase (SAPK) or nuclear factor kappa-B (NF-κB) signaling pathways. Activating these signaling pathways can modulate the process of autophagy, thereby affecting cellular survival and death (Qin et al., 2022; Pang et al., 2023b). Research indicates that integrin signaling can positively modulate neural plasticity by enhancing neuronal autophagy (**Figure 6** and **Additional Table 8**). Following IS, endogenous osteopontin (OPN) and Beclin1 are increased in the perilesional area of stroke patients and rodents. Treatment with OPN not only elevates the protein levels of integrin β1 but also promotes the expression of the autophagy-related proteins Beclin1 and microtubule-associated protein 1 light chain 3 (LC3) in the perilesional area. Furthermore, the neuroplasticity and autophagy induced by OPN can be blocked by integrin antagonists, suggesting that OPN may enhance neural plasticity through autophagy, suggesting a novel therapeutic strategy for IS (Liao et al., 2022).

*Role of the integrin family in vascular endothelial cell dysfunction in ischemic stroke*

**Figure 7:**
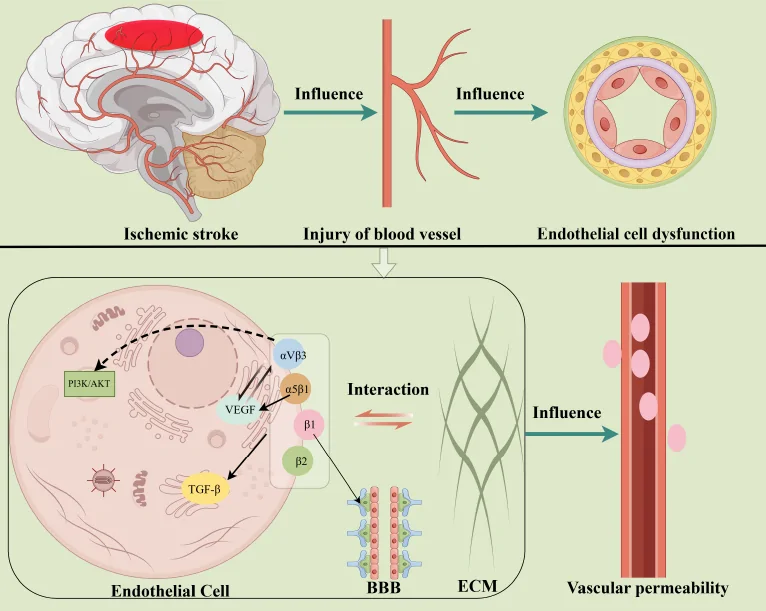
Members of the integrin family regulate the process of IS by participating in vascular endothelial cell dysfunction. Apoptosis of endothelial cells can be modulated by integrins through the PI3K/Akt pathway, influencing cell survival. Post-IS angiogenesis is crucial for functional recovery, with integrins such as αvβ3 and β1 playing roles in promoting vascular repair and neuroremodeling. Integrin β1 is particularly critical for angiogenesis and neurovascular remodeling. Inhibitors of integrins may improve endothelial function and reduce complications, presenting a potential therapeutic target for the treatment of IS. Created with Figdraw 2.0 (export code: APATA143fb). AKT: Protein kinase B; BBB: blood–brain barrier; ECM: extracellular matrix; IS: ischemic stroke; PI3K: phosphoinositide-3 kinase; TGF-β: transforming growth factor β; VEGF: vascular endothelial growth factor.

Vascular endothelial cells may undergo apoptosis in IS due to hypoxia and nutrient deprivation. Integrins can affect the survival and apoptosis of vascular endothelial cells by regulating survival signaling pathways (Pang et al., 2023b). Following IS, vascular repair and regeneration are crucial for the restoration of cerebral blood flow. Postischemic angiogenesis can facilitate neuronal remodeling and is believed to contribute to functional recovery. This is attributed to the role of newly formed vessels postischemia in guiding sprouting axons through the signaling of VEGF and laminin/β1-integrin (Hatakeyama et al., 2020). Integrins participate in the vascular repair process by promoting angiogenesis and the migration and proliferation of vascular endothelial cells (Aman and Margadant, 2023). Lathia et al. (2010) reported that β1 integrin expression, which is localized exclusively to blood vessels, is essential for endothelial cell migration, proliferation, and angiogenesis. β1 integrin signaling is involved in the formation and restoration of the vasculature following IS and plays a significant role in neurovascular remodeling and functional outcomes post-IS. Edwards et al. (2020) demonstrated that endothelial cell β1 integrin receptors can directly contribute to BBB dysfunction. Following the administration of an integrin α5β1 inhibitor, a significant reduction in infarct volume, edema, and functional deficits was observed in mice. Lee et al. (2011) discovered that the naturally occurring perlecan ECM fragment, domain V, possesses neuroprotective properties and enhances poststroke angiogenesis. The administration of domain V following IS increases VEGF levels through a mechanism involving α5β1 integrin on brain endothelial cells; subsequently, the neuroprotective and angiogenic effects of domain V are mediated through VEGFR. Additionally, a study has shown that both the α subunits of β2 integrins contribute to brain injury and leukocyte‒vascular wall interactions associated with transient focal cerebral ischemia (Arumugam et al., 2004). Owing to their role in vascular endothelial dysfunction, integrins are considered potential therapeutic targets (**Figure 7** and **Additional Table 8**). Treatment strategies targeting integrins, such as integrin inhibitors, may help improve vascular endothelial function and reduce vascular complications.

#### Role of integrins in hemorrhagic stroke

HS arises from the rupture of cerebral blood vessels, leading to the extravasation of blood into the brain parenchyma (intracerebral hemorrhage) or the subarachnoid space (subarachnoid hemorrhage) (Tan et al., 2024; Li et al., 2025a). Although its incidence is lower than that of IS, HS is associated with higher morbidity and mortality, partly due to the sudden elevation in intracranial pressure and the neurotoxicity of blood components (van Asch et al., 2010; Cordonnier et al., 2018). The ensuing pathophysiology involves acute increases in intracranial pressure, localized tissue ischemia, excitotoxicity, inflammation, oxidative stress, and apoptosis (Wang, 2010; Keep et al., 2012). While integrins have been extensively studied in IS, growing evidence suggests that they also play a critical role in HS, influencing platelet function, inflammatory cell recruitment, and BBB integrity.

**Figure 8:**
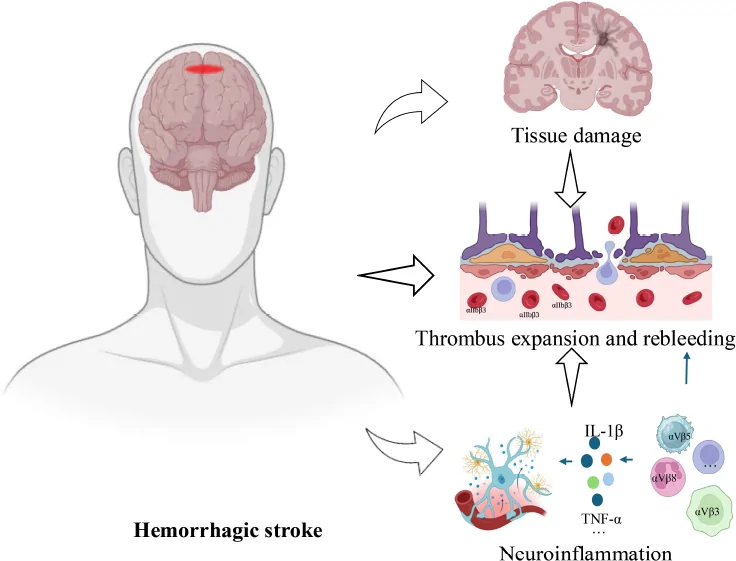
Effect of integrins on hemorrhagic stroke. Integrins influence platelet function, immune cell recruitment, and blood-brain barrier integrity. Modulating integrin αIIbβ3 may control thrombus expansion and rebleeding. Targeting β2 integrins reduces neuroinflammation, and αVβ3 integrins, involved in repair, may protect vascular integrity and BBB. These integrins could be therapeutic targets for hemorrhagic stroke. BBB: Blood–brain barrier; IL-1β: interleukin-1β; TNF-α: tumor necrosis factor-α.

In the context of HS, modulating αIIbβ3 activity may help control thrombus expansion and prevent rebleeding (Montecino-Garrido et al., 2024; Oshinowo et al., 2024). While antiplatelet or anticoagulant therapies targeting αIIbβ3 have been widely employed in cardiovascular diseases, their use in HS remains controversial due to the potential risk of exacerbating hemorrhage. However, precision strategies targeting platelet activity at the site of hemorrhage may offer therapeutic benefits. Infiltrating leukocytes release pro-inflammatory cytokines, proteases, and ROS, exacerbating edema and neuronal injury (Urday et al., 2016). Pharmacological or genetic blockade of β2 integrins has demonstrated neuroprotective potential in experimental hemorrhagic models, reducing neuroinflammation and tissue damage (Harjunpää et al., 2024). Integrin αV has been implicated in inflammatory responses and tissue repair after intracerebral hemorrhage (McCarty et al., 2002). Studies suggest that αV-containing integrins may support vascular integrity and mitigate BBB disruption in hemorrhagic lesions, similar to their roles in IS (McCarty et al., 2005; Wang et al., 2022). Thus, targeting αVβ3 may represent a strategy to balance inflammatory cell recruitment and tissue repair (**Figure 8** and **Additional Table 8**).

### Role of the integrin family in Alzheimer’s disease

AD is a progressive, age-related neurodegenerative disorder and the leading cause of dementia worldwide (Memudu et al., 2024; Vejandla et al., 2024). Clinically, AD is characterized by deficits in cognition, memory, and executive function, ultimately impairing an individual’s ability to perform daily activities (Ghayedi et al., 2024). Pathologically, AD is marked by extracellular Aβ plaque deposition, intracellular accumulation of hyperphosphorylated tau in neurofibrillary tangles, widespread neuroinflammation, and synaptic/neural loss (Selkoe and Hardy, 2016; DeTure and Dickson, 2019). Integrins have emerged as critical regulators in multiple aspects of AD pathogenesis, including Aβ production/clearance, neuroinflammation, and synaptic maintenance (Cappellano et al., 2013; Dominguez, 2019). Understanding the role of integrins in AD may pave the way for novel therapeutic interventions aimed at preventing or slowing disease progression.

Microglia are the resident immune cells of the CNS and play a pivotal role in surveillance and homeostasis maintenance within the brain (Kent and Miron, 2024; Muzio and Perego, 2024). One of their critical functions is the phagocytic clearance of Aβ and other cellular debris (Almalki and Almujri, 2024; Deng et al., 2024). Microglia express integrins such as αMβ2 and αVβ3, which facilitate Aβ aggregate adhesion, migration, and uptake (Wennström and Nielsen, 2012). Disruption or downregulation of these integrins impairs Aβ clearance, leading to increased plaque burden. Conversely, enhancing integrin function has been shown to promote Aβ fibril uptake by microglia, potentially delaying disease progression (Wennström and Nielsen, 2012). While integrins are primarily implicated in Aβ clearance, emerging evidence suggests they may also influence amyloid precursor protein processing and amyloidogenesis (Dourlen et al., 2019; Pfundstein et al., 2022). For instance, β1 integrin signaling has been reported to modulate amyloid precursor protein trafficking and secretase activity, though the underlying mechanisms remain incompletely understood (Koenigsknecht and Landreth, 2004). These findings highlight integrin-centered strategies as potential therapeutic avenues to mitigate pathological Aβ accumulation in AD.

A defining feature of AD is chronic neuroinflammation, predominantly driven by the robust activation of microglia and astrocytes surrounding amyloid plaques (Heppner et al., 2015). While the initial glial response may be protective—aimed at containing or clearing plaques—prolonged activation can lead to excessive release of pro-inflammatory mediators, such as TNF-α, IL-1β, and IL-6, thereby exacerbating neuronal damage (Akiyama et al., 2000). Astrocyte-expressed αV integrins, including αVβ5 and αVβ8, regulate the activation of latent TGF-β, modulating the astrocytic and local microenvironment toward either pro-inflammatory or anti-inflammatory states (McCarty, 2020; Kim et al., 2023; Shen et al., 2024). Dysregulation of this pathway may shift the balance toward deleterious neuroinflammation, thereby promoting plaque expansion and neuronal injury. β2 integrin subunits on microglia, such as αMβ2, play a crucial role in guiding microglial migration and facilitating Aβ plaque phagocytosis (Ahn and Park, 2025). Persistent overactivation of microglia or aberrant integrin signaling may exacerbate neuroinflammatory cascades and impair reparative processes (Botella Lucena and Heneka, 2024). Thus, integrins function as critical molecular switches that can either suppress or amplify neuroinflammatory responses, making them promising therapeutic targets for mitigating inflammation-driven AD pathology.

**Figure 9:**
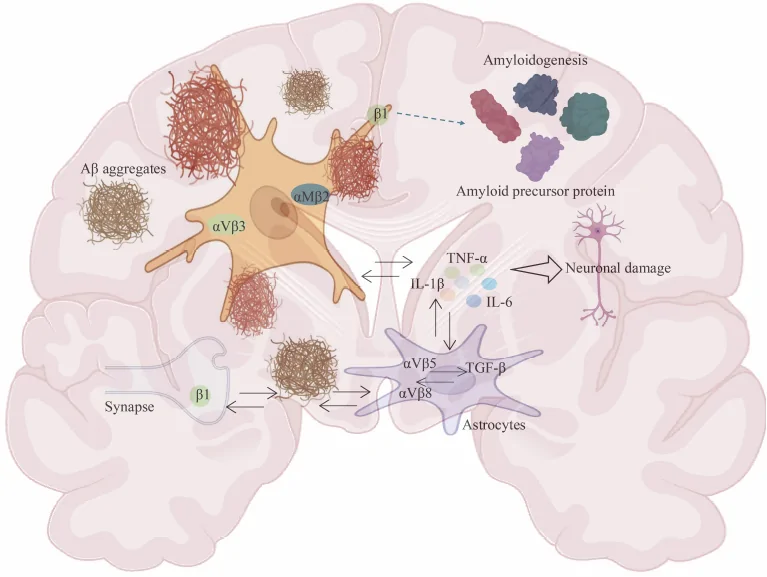
Effect of integrins on Alzheimer’s disease. Integrins have potentials for Aβ clearance, neuroinflammation, and synaptic function. Microglia utilize integrins to facilitate the uptake of Aβ, and alterations in integrin function may impact disease progression. Additionally, integrins modulate neuroinflammation and synaptic plasticity, positioning them as potential therapeutic targets for Alzheimer’s disease treatment. Aβ: Amyloid-beta; IL: interleukin; TGF-β: transforming growth factor β; TNF-α: tumor necrosis factor-α.

Synaptic loss in AD represents a primary correlate of cognitive decline and memory impairment (Terry et al., 1991). β1 integrin, which is widely expressed at both pre- and postsynaptic sites, plays a pivotal role in synaptic structure, stability, and plasticity (Yan and Cui, 2023). In memory impairment model, Aβ oligomers can directly or indirectly interact with β1 integrins at synapses, leading to morphological alterations, dendritic spine retraction, and impairment of long-term potentiation (Biose et al., 2023). This disruption of integrin signaling compromises synaptic vesicle clustering, receptor trafficking, and the actin cytoskeleton remodeling necessary for sustained synaptic transmission. However, experimental strategies aimed at stabilizing or upregulating β1 integrin expression has shown promising effects in rescuing synaptic deficits. Enhancing β1 integrin signaling has been demonstrated to restore dendritic spine density, and improve synaptic plasticity (Huang et al., 2006). Thus, targeting β1 integrin-associated pathways may represent a crucial strategy for preserving or restoring synaptic function in AD-affected brains (**Figure 9** and **Additional Table 8**).

### Role of the integrin family in Parkinson’s disease

PD is an insidious neurodegenerative disorder characterized by the progressive loss of dopaminergic neurons in the substantia nigra pars compacta (Bhandari et al., 2024; He et al., 2024a), leading to hallmark motor impairments, including resting tremor, rigidity, and bradykinesia, as well as a spectrum of non-motor symptoms such as depression, anxiety, cognitive decline, and sleep disturbances (Bloem et al., 2021). The pathological hallmark of PD is the misfolded aggregation of SNCA within Lewy bodies, which precipitates neuronal dysfunction and eventual cell death (Bayati and McPherson, 2024; Leak et al., 2024). While conventional research has primarily focused on the biology of dopaminergic neurons, emerging evidence implicates integrins in neuroinflammatory and neuroprotective pathways associated with PD pathogenesis (Chapman and Sorg, 2024; Cui et al., 2024a). The etiology of PD is multifactorial, involving genetic predisposition (e.g., mutations in SNCA, leucine-rich repeat kinase 2 (LRRK2), and parkin RBR E3 ubiquitin protein ligase (PARK2)), environmental exposures (e.g., pesticides and heavy metals), and neuroinflammatory processes in the midbrain (Dorsey and Bloem, 2018; Guessoum et al., 2024; Tenchov et al., 2025). The degeneration of substantia nigra pars compacta dopaminergic neurons disrupts the output of the nigrostriatal pathway, leading to alterations in basal ganglia circuitry that manifest as the core motor dysfunctions of PD (Fishman-Jacob and Youdim, 2024). Non-motor symptoms, including cognitive impairment, autonomic dysfunction, and mood disturbances, further contribute to the substantial reduction in patients’ quality of life (Chaudhuri et al., 2006). Increasing evidence suggests that neuroinflammation, driven by activated microglia, reactive astrocytosis, and peripheral immune cell infiltration, accelerates disease progression through the release of pro-inflammatory cytokines and ROS (Joers et al., 2017).

**Figure 10:**
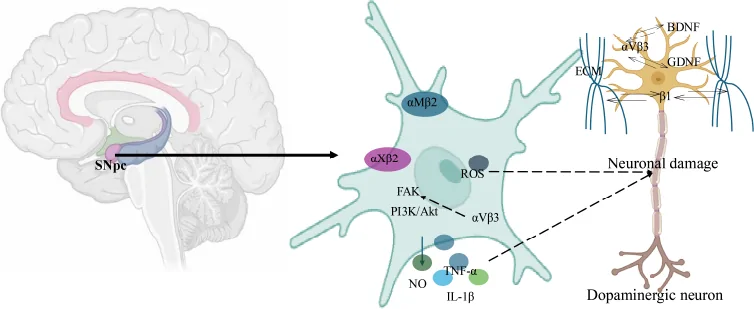
Effect of integrins on Parkinson’s disease. Integrins impact neuroinflammation and dopaminergic neuron survival. β2 integrins exacerbate oxidative stress, β1 integrins interact with the ECM for support, and αVβ3 integrins are crucial for neuronal survival. Integrin signaling represents a potential therapeutic target for Parkinson’s disease. AKT: Protein kinase B; BDNF: brain-derived neurotrophic factor; ECM: extracellular matrix; FAK: focal adhesion kinase; GDNF: glial cell line-derived neurotrophic factor; IL-1β: interleukin-1β; NO: nitrogen monoxide; PI3K: phosphoinositide-3 kinase; ROS: reactive oxygen species; SNpc: substantia nigra pars compacta; TNF-α: tumor necrosis factor-α.

Despite being relatively understudied in PD compared to other neurological disorders such as AD and MS, several lines of evidence support a role for integrin-mediated signaling in the maintenance and vulnerability of dopaminergic neurons. In PD, microglia within the substantia nigra pars compacta may become hyperactivated (Bej et al., 2024; Harackiewicz and Grembecka, 2024), expressing high levels of β2 integrins (e.g., αMβ2, αXβ2), which facilitate microglial adhesion and migration toward degenerating neurons or α-synuclein inclusions, where they may exacerbate oxidative stress and release pro-inflammatory cytokines (Surguchev and Surguchov, 2019; Juul-Madsen et al., 2020; Li et al., 2021). Chronic inflammation, supported by integrin-expressing glial cells, may amplify neuronal damage by excessive production of nitric oxide (NO) and ROS, thereby accelerating α-synuclein pathology (Hirsch and Hunot, 2009). Astrocyte-secreted ECM proteins interact with β1 integrins on dopaminergic neurons, providing trophic support and contributing to synaptic stability. In PD, astrocytic dysfunction or alterations in integrin signaling may compromise neuronal metabolic and structural support, exacerbating neurodegeneration (Pekny and Pekna, 2014). Changes in ECM composition, such as increased deposition of fibronectin or laminin, can modulate β1 integrin-dependent neuronal survival pathways (Schweitzer, 2015), which may also influence neurite outgrowth and synapse maintenance in the PD setting. αVβ3 integrin interacts with RGD-containing ECM proteins (e.g., OPN, vitronectin) and growth factors to activate downstream pathways critical for neuronal survival and plasticity, including FAK and PI3K/Akt signaling (Lowell and Mayadas, 2012; Sohrabi, 2021). In dopaminergic neurons, integrin may cooperate with BDNF to enhance cell survival under stress conditions (Tang et al., 2023a). Thus, disruption of αVβ3 activity may increase neuronal susceptibility to PD-related insults, further highlighting the role of integrin signaling in the pathophysiology of PD (**Figure 10** and **Additional Table 8**).

### Role of the integrin family in multiple sclerosis

MS is a chronic, immune-mediated demyelinating disorder of the CNS and a leading cause of non-traumatic neurological disability in young adults worldwide (Filippi et al., 2018; Dobson and Giovannoni, 2019). MS is characterized by multifocal inflammation, demyelination, and axonal damage, presenting in various clinical forms, with the most common being the relapsing-remitting phenotype, which may eventually transition to secondary progressive MS (Perdaens and van Pesch, 2024; Lorenzut et al., 2025). The pathogenesis of MS is primarily driven by an aberrant immune response against myelin components (Dobson and Giovannoni, 2019). Peripheral immune cells, particularly T lymphocytes, become activated and cross the BBB. Upon entry into the CNS, these cells secrete pro-inflammatory cytokines, recruiting macrophages and microglia, which further contribute to demyelination, gliosis, and subsequent axonal degeneration (Filippi et al., 2018; Dobson and Giovannoni, 2019; Al-Ogla et al., 2024; Xu et al., 2024). While genetic predisposition and environmental factors, such as viral infections and vitamin D deficiency, appear to modulate individual susceptibility to MS, its precise etiology remains incompletely understood (Reich et al., 2018; Belbasis et al., 2020). Pathologically, MS lesions exhibit inflammatory infiltrates composed of T cells, B cells, macrophages, and microglia, which differentially contribute to demyelination and remyelination. Recurrent episodes of demyelination may lead to progressive neurodegeneration and cumulative disability over time (Leary et al., 2024; Stys et al., 2024; Woo et al., 2024).

A breakthrough in understanding the pathogenesis of MS was the identification of α4 integrin (α4β1), also known as very late antigen-4, on T cells and its interaction with vascular cell adhesion molecule-1 on endothelial cells (Yednock et al., 1992; Baron et al., 1993). This integrin-mediated binding is critical for T cell adhesion to CNS vasculature and subsequent migration into the CNS parenchyma. The α4–vascular cell adhesion molecule-1 interaction enables T cells to roll and firmly adhere to activated CNS endothelium (Laschinger and Engelhardt, 2000; Vajkoczy et al., 2001), while disruption of α4 function significantly reduces T cell infiltration and CNS lesion formation, thereby attenuating demyelination and inflammation (Bierhansl et al., 2022; Heng et al., 2022; Liu et al., 2022). Natalizumab (Tysabri), a humanized mAb targeting the α4 subunit, effectively blocks the α4β1–vascular cell adhesion molecule-1 interaction. Clinically approved for relapsing forms of MS, natalizumab substantially reduces relapse rates and the formation of new lesions by preventing immune cell transmigration across the BBB (Polman et al., 2006). Despite its efficacy, natalizumab carries a risk of progressive multifocal leukoencephalopathy associated with John Cunningham virus infection, underscoring the need for rigorous patient monitoring. The success of natalizumab highlights the critical role of integrin-dependent immune cell trafficking in MS and has spurred interest in developing additional integrin-targeted therapies.

**Figure 11:**
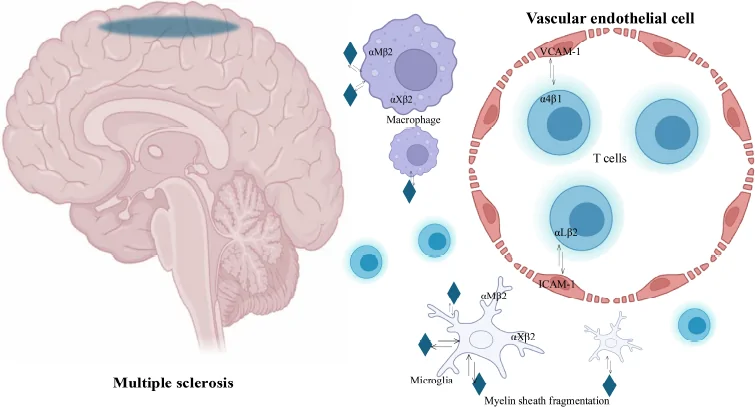
Effect of integrins on multiple sclerosis. MS is characterized by demyelination and axonal damage resulting from immune cells traversing the blood-brain barrier. The interaction of α4β1 integrin with VCAM-1 facilitates T cell migration into the central nervous system, triggering neuroinflammation. β2 integrins are also involved in the recruitment of immune cells and phagocytosis of myelin debris, while β1 integrins are crucial for the function of oligodendrocyte precursor cells and remyelination. Targeting these integrins may offer novel therapeutic strategies for MS. MS: Multiple sclerosis; VCAM-1: vascular cell adhesion molecule 1.

β2 integrins (CD18 family), including αMβ2 and αXβ2, are expressed on activated microglia and infiltrating macrophages, where they regulate the phagocytosis of myelin debris (Raivich and Banati, 2004; Ong, 2017). Dysregulated or excessive activation of these integrins may exacerbate demyelination and tissue damage, particularly when myelin clearance becomes excessive or triggers chronic inflammation (Németh et al., 2016; Iaia, 2021; Rossi et al., 2021). αLβ2 (lymphocyte function-associated antigen-1) plays a pivotal role in T cell extravasation, facilitating T lymphocyte migration across the BBB via interactions with intercellular adhesion molecule-1 (Amoriello et al., 2024; Zhang et al., 2025). Targeting αLβ2 may selectively reduce T cell infiltration, akin to α4 integrin blockade, but with potentially distinct immunological specificity. Moreover, β1 integrins in OPCs are essential for remyelination, regulating OPC migration, proliferation, and differentiation within demyelinated lesions (Neili et al., 2024; Yamada et al., 2025). Theoretically, enhancing β1 integrin signaling in OPCs could promote endogenous remyelination, potentially mitigating progressive axonal loss and disability (**Figure 11** and **Additional Table 8**).

### Role of the integrin family in other emerging neurological conditions

In emerging neurological diseases, integrins play a pivotal role and show great potential. In traumatic brain injury (TBI), post-trauma integrin expression and activation in CNS cells (neurons, astrocytes, microglia, and endothelial cells) change significantly, involving multiple key processes. Integrins modulate neuroinflammation by regulating immune cell recruitment and activation. They maintain BBB integrity via mediating endothelial cell-ECM adhesion; integrin signal disruption can cause BBB breakdown and increased permeability, leading to neural damage. Also, integrins promote cell migration and adhesion, crucial for tissue repair, but contribute to glial scar formation, which may hinder axonal regeneration. For example, Tang et al. (2023b) found tissue inhibitor of metalloproteinase-2 reduces neurodeficits and BBB leakage in TBI mice by interacting with α3β1 integrin on endothelial cells, inhibiting Src-dependent VE-cadherin phosphorylation. Li et al. (2025b) showed blocking β2 integrin improves neurofunction in TBI mice, reducing BBB permeability, oxidative stress, and inflammatory mediator release, while enhancing cerebral perfusion. Dean et al. (2024) demonstrated that blocking fibrinogen-CD11b interaction decreases monocyte recruitment and inflammation/ROS pathway activation in microglia and macrophages post-TBI, alleviating oxidative damage and cortical loss. Schiweck et al. (2021) revealed that post-injury upregulation of Drebrin in astrocytes is vital for scarring and astrocyte reactivity. Drebrin loss impairs scarring and causes excessive neurodegeneration, affecting β1-integrin transport. Collectively, these studies highlight integrins’ diverse roles in TBI pathogenesis. Their activation can be double-edged: appropriate activation aids repair, but overexpression or dysfunction exacerbates injury (**Additional Table 8**).

In neurodevelopmental disorders, integrins are essential for neuronal migration, axon navigation, and synapse formation. Their interaction with ECM components (like laminin, fibronectin, and collagen) guides neurons to correct positions, and disruptions in this process can cause neuronal misplacement and circuit dysfunction. Integrin signaling also partially regulates synaptic stability and plasticity; its dysfunction may lead to cognitive and behavioral deficits. Additionally, integrins help growing axons navigate by interacting with various ECM molecules, ensuring proper brain connectivity. Dysfunction here can cause connectivity issues, contributing to certain neurodevelopmental disorders. Fuma et al. (2025) found prenatal inflammation damages early CD11c (αX integrin)-positive microglia, hindering myelination in neurodevelopmental disorders, with a lack of high-CD11c microglia observed in affected children. Amezquita et al. (2024) demonstrated integrin adhesion axes inhibit E3 ubiquitin-protein ligase RPM-1 ubiquitin ligase signaling, regulating growth cone and axon development. Bilches Medinas et al. (2022) showed mutant protein disulfide isomerase A3 disrupts cell adhesion and actin cytoskeleton dynamics, linked to altered integrin biogenesis and reduced neurogenesis, ultimately causing neurodevelopmental defects via endoplasmic reticulum protein homeostasis disruption. Arnold et al. (2019) showed conditional αVβ8 deficiency in the CNS prevents normal microglia maturation, resulting in unique neurodevelopmental syndromes characterized by oligodendrocyte maturation arrest, interneuron loss, and spastic motor dysfunction. These studies collectively highlight integrins’ crucial role in neurodevelopmental disorders, where their dysfunction can lead to various neurodevelopmental issues, making them a key research focus (**Additional Table 8**).

Integrins, pivotal in injury response and development, are promising therapeutic targets. Modulating their signaling could alleviate TBI-induced inflammation, stabilize the BBB promote regeneration, reduce glial scarring, and correct neuronal migration and synapse formation abnormalities, thereby lessening the impact of neurodevelopmental disorders. Current research focuses on uncovering the precise molecular mechanisms of integrin involvement in these processes. A deeper understanding of these pathways will aid in developing new interventions for TBI recovery and neurodevelopmental disorders. As key regulators of CNS structure and function, integrin dysfunctions are closely linked to acute TBI responses and long-term neurodevelopmental abnormalities. Further studying integrin signaling pathways could offer new targeted therapy strategies for these complex diseases.

## *In Vivo* Imaging of Integrin Function in Neurological Disorders

Recent advances in *in vivo* imaging technologies have significantly enhanced our ability to monitor integrin-mediated signaling, offering new prospects for the diagnosis and treatment of neurological disorders. Integrins, as pivotal molecules involved in cellular interactions with the ECM and between cells, play essential roles in various neurological diseases, including neuroinflammation, neurodegenerative disorders, and brain tumors. Real-time visualization of integrin activity not only facilitates precise diagnosis but also supports the development of more targeted therapeutic approaches. Modern *in vivo* imaging techniques, such as positron emission tomography (PET), magnetic resonance imaging (MRI), and optical imaging, have become indispensable tools for studying the role of integrins in pathological processes, enabling researchers to gain deeper insights into disease mechanisms.

Integrin-targeted imaging probes have emerged as powerful tools in the study of the pathophysiology of neurological disorders, providing valuable insights into disease progression and evaluating the efficacy of immunomodulatory therapies. In the past decade, significant progress has been made in imaging technologies for monitoring integrin function. PET, utilizing radiolabeled ligands or antibodies that specifically bind to integrin receptors, has enabled real-time tracking of integrin expression and activity. For example, Beer et al. (2011) described PET imaging with αvβ3 integrin-specific probes to visualize brain tumors *in vivo*, allowing dynamic monitoring of tumor progression and evaluation of integrin-targeted therapies. Additionally, PET imaging of α6 integrins has been used to assess the intensity of brain sites producing focal radioactivity (Zhang et al., 2022a). While MRI itself does not directly measure integrin function, it can be combined with integrin-targeted contrast agents to precisely localize integrin-rich areas, such as tumor borders or regions of neuroinflammation, thereby monitoring tumor progression and therapy outcomes. Optical imaging technologies, particularly fluorescence microscopy and bioluminescence imaging, offer high spatial resolution and are highly suited for monitoring integrin-mediated events at the cellular level, especially in preclinical models of neurological diseases.

Despite the promising applications of integrin-targeted imaging, several challenges remain. One key hurdle is the development of highly specific and high-affinity probes. Given that many integrin subtypes are expressed in various cell types, cross-reactivity of imaging probes can lead to nonspecific binding, which compromises signal accuracy. Furthermore, although PET and MRI excel in monitoring large-scale changes in integrin expression, their spatial resolution is limited, especially when detecting small lesions or subtle alterations in integrin activity. Future developments in hybrid imaging technologies, such as PET/MRI or PET/CT, may help enhance both spatial resolution and sensitivity. Another challenge is the translation of these imaging techniques from preclinical studies to clinical settings. The development of safe and effective radiolabeled agents for use in human patients is critical for the clinical success of integrin-targeted imaging. Regulatory hurdles, including safety and efficacy evaluations, must be addressed to ensure these imaging technologies can be routinely applied in clinical practice. Looking ahead, the continued advancement of integrin-targeted imaging, coupled with its integration with emerging technologies such as gene therapy, stem cell therapies, and personalized medicine, holds promise for providing more precise treatment strategies for neurological diseases. Integrin-targeted imaging will not only play a crucial role in disease diagnosis but also provide robust support for monitoring the effectiveness of integrin-targeted therapies. As technological advancements continue and interdisciplinary collaborations deepen, integrin-targeted imaging is poised to play a pivotal role in the clinical management of neurological disorders, unlocking its full potential in therapeutic applications.

## Integrin-targeted Small Molecule and Biologic Therapies in Neurological Disorders

### Integrins as biomarkers for neurological disease

Integrins play a crucial role in a variety of cellular functions that are integral to the progression of neurological diseases. Their dysregulation can profoundly impact disease mechanisms, including neuroinflammation, neuronal survival, axonal regeneration, and the integrity of the BBB. As such, integrin expression profiles and functional status provide valuable biomarkers for predicting disease progression, prognosis, and therapeutic response. In neurological diseases such as AD, PD, and MS, specific integrins serve as indicators of disease state. For example, in AD, the upregulation of integrin β3 in astrocytes has been associated with increased neuroinflammation and neuronal damage (Ivanova et al., 2024). In PD, integrin α4β1 is involved in the migration of immune cells into the brain, contributing to neurodegeneration (Yuan et al., 2024b). Thus, detecting changes in integrin expression or activation could provide insights into disease activity and therapeutic potential. Recent advances in liquid biopsy technologies have enabled the detection of circulating integrins and their fragments in blood or cerebrospinal fluid as potential biomarkers for neurological diseases. For instance, changes in levels of soluble integrins such as αvβ3 and α5 have been linked to BBB permeability, neuroinflammation and neuronal damage in neurological disorders (Ruzha et al., 2022; Cui et al., 2024a). These integrin-related biomarkers offer promise in predicting disease progression and monitoring the effectiveness of integrin-targeted therapies. Furthermore, genetic mutations or polymorphisms in integrin genes can influence their functional activity and impact individual responses to treatment. Single nucleotide polymorphisms in integrin genes, such as the *ITGB3* gene encoding integrin β3, have been associated with altered integrin signaling and variations in disease susceptibility in conditions like autism (Schuch et al., 2014). Identifying genetic variants in patients may help predict their response to integrin-targeted therapies, thus facilitating more precise and personalized treatment strategies.

### Genetic and molecular profiling for optimizing neurological treatments

The integration of genetic, transcriptomic, and proteomic profiling into the clinical management of neurological diseases holds the potential to significantly enhance the efficacy of integrin-targeted therapies. By analyzing genomic alterations in genes encoding integrins and associated signaling molecules, clinicians can better understand how these factors influence disease progression and treatment response. For instance, in MS, genetic variations in ITGB1 and ITGA4, which encode integrin β1 and α4 subunits respectively, may affect immune cell migration and the patient’s response to therapies targeting these pathways, such as natalizumab (Clarelli et al., 2024). Genomic analysis allows for the prediction of patient responses to α4β1-targeted treatments, thereby optimizing therapeutic strategies and minimizing adverse effects. Beyond genomics, transcriptomic and proteomic analyses offer valuable insights into the expression of integrin receptors, downstream signaling molecules, and other key regulators involved in neuroinflammation and neurodegeneration. For example, in AD, profiling microglial activation markers alongside integrin expression can identify which patients are likely to benefit from therapies targeting integrin αvβ3 (Qiu et al., 2023). Likewise, proteomic profiling can identify soluble integrins or integrin-related proteins as potential biomarkers for predicting therapeutic responses. By combining integrin profiling with genetic, transcriptomic, and proteomic data, clinicians can develop personalized treatment strategies tailored to the individual molecular profile of each patient. For instance, patients with high αvβ3 expression may benefit from FAK inhibitors (Che et al., 2021; Zhang et al., 2022b), while those with elevated α4β1 expression could respond more favorably to natalizumab (Chaves et al., 2024; Cardle et al., 2025). These personalized strategies aim not only to optimize therapeutic efficacy but also to minimize the risk of side effects by precisely targeting treatments to the patient’s specific molecular characteristics.

### Strategies for targeting integrins

Growing evidence highlights the role of integrins as key mediators of inflammation, cell survival, and angiogenesis across multiple neurological disorders. These insights have driven the development of integrin-targeted therapeutics, including mAbs, small molecules, and peptides, each with distinct mechanisms of action and clinical potential. Among integrin-targeting strategies, mAbs exhibit high specificity for integrin subunits, preventing ligand binding and thereby reducing pathological cell adhesion or infiltration. For example, natalizumab targets α4 integrin to inhibit T cell migration across the BBB in relapsing MS (Polman et al., 2006), while abciximab binds to αIIbβ3 integrin on platelets and is primarily used as an antiplatelet agent in cardiovascular interventions (Lee et al., 2024b). Peptides and peptidomimetic inhibitors, such as cyclic RGD motifs, competitively block integrin interactions with ECM proteins, attenuating downstream pro-inflammatory or pro-angiogenic signaling (Schussler et al., 2022). Notably, cRGDfV, a cyclic RGD peptide, selectively inhibits αVβ3 integrin and has been shown to reduce angiogenesis and inflammation in rodent models of IS (Bi and Yi, 2014; Mousa et al., 2017). Small-molecule inhibitors, which can allosterically modulate integrin conformation or directly compete with ECM ligands at the integrin binding site, offer advantages over mAbs, including oral bioavailability and simpler pharmacokinetics. For instance, ATN-161, an α5β1 antagonist, has been investigated in preclinical stroke models, demonstrating neuroprotective and anti-inflammatory properties (Edwards, 2019). Nanoparticle-based drug delivery strategies further enhance integrin-targeted therapy by conjugating integrin-binding ligands or peptides (e.g., RGD) to nanocarriers such as liposomes and polymeric nanoparticles, enabling selective targeting of integrins that are overexpressed in pathological conditions (Wang et al., 2010; Zhao et al., 2020). This approach improves drug specificity, protects encapsulated therapeutics from degradation, and potentially circumvents BBB efflux mechanisms, thereby optimizing therapeutic efficacy.

### Clinical and preclinical trials overview

Integrin-targeted therapies have demonstrated multifaceted potential in the treatment of neurological disorders. In IS, preclinical studies of α5β1 inhibitors (e.g., ATN-161) and αVβ3 inhibitors (e.g., cRGD peptides) have shown efficacy in reducing leukocyte infiltration, mitigating BBB disruption, and decreasing infarct volume (Bi and Yi, 2014; Edwards, 2019). Junghans et al. (2002) studied whether tirofiban, a selective platelet aggregation inhibitor, could prevent ischemic tissue expansion in acute ischemic stroke patients. They found that treated patients had smaller infarct volumes than matched controls with similar initial perfusion deficits. They concluded that GP IIb/IIIa antagonists show therapeutic potential in acute stroke treatment. Although clinical trials remain limited, early data suggest their feasibility in terms of safety and efficacy. In HS, αIIbβ3-targeted strategies aimed at stabilizing thrombi have highlighted potential benefits in preventing hematoma expansion or hemorrhagic transformation (Montecino-Garrido et al., 2024; Oshinowo et al., 2024); however, robust clinical trials in this context remain scarce. In AD, emerging strategies focus on modulating αMβ2 or αVβ3 integrins to enhance microglial clearance of Aβ, while promoting β1 integrin activity to preserve synaptic integrity (Ivanova et al., 2024; Ahn and Park, 2025). Most integrin-targeted approaches for AD remain in early preclinical or proof-of-concept stages. In PD, αVβ3 is under investigation, with therapeutic efforts directed at suppressing microglial overactivation and enhancing neuronal survival (Li et al., 2022, 2023a), though clinical trials have yet to be realized. In MS, natalizumab (Tysabri) remains the primary integrin-targeted therapy, effectively reducing relapse rates and new lesion formation in relapsing MS by blocking α4 integrin (Polman et al., 2006). Clinical trial results from Foley et al. (2024) indicate that natalizumab levels and α4-integrin saturation in patients have some predictive value for MS disease activity and relapses, though not definitive. While the authors advise cautious interpretation of α4-integrin saturation measurements, their utility is evident. Looking ahead, advancements in artificial intelligence and technology may enable multi-marker diagnostics or methods targeting integrin downstream effects, potentially enhancing diagnostic and therapeutic outcomes. Meanwhile, investigations into other integrin subunits, such as β2 integrins, seek to further optimize therapeutic efficacy while minimizing risks, including progressive multifocal leukoencephalopathy. These advancements underscore the broad therapeutic potential of integrin-targeted therapies across various neurological disorders, paving the way for further translational and clinical development.

### Opportunities and challenges

Despite the promising advancements in integrin-targeted therapies, several critical challenges remain. Firstly, the widespread distribution of integrin subunits across various tissues raises concerns regarding off-target effects, such as coagulation disorders and immunosuppression. To optimize safety, tailoring delivery or expression specifically to the CNS is crucial. Secondly, the redundancy of integrin signaling and shared ECM ligands may attenuate the long-term efficacy of targeting a single integrin subunit. Consequently, combination approaches or multi-pronged strategies may be required to achieve durable therapeutic responses. Additionally, biological therapies (e.g., mAbs, peptides) often face challenges in penetrating the BBB. Advanced strategies such as nanoparticle carriers, intracerebral injection, or engineered delivery systems may enhance CNS drug bioavailability while minimizing systemic effects. Prolonged integrin inhibition also carries risks of opportunistic infections (e.g., progressive multifocal leukoencephalopathy) and hemorrhagic complications. Strict patient selection, biomarker-driven approaches, and vigilant monitoring could mitigate these risks. In acute neurological disorders (e.g., IS), the narrow therapeutic window makes timing a critical factor, and long-term integrin blockade may disrupt essential processes such as tissue remodeling and immune surveillance. In the future, with the development of artificial intelligence and new technologies, multi-marker diagnostics and targeted therapies could be applied to clinical research and treatment, potentially offering better therapeutic effects. Overall, integrin-targeted therapies hold considerable potential for addressing neuroinflammation, BBB dysfunction, and neuronal loss across various CNS pathologies. Future efforts should focus on enhancing target specificity, refining drug delivery, and balancing immune modulation with patient safety, ultimately expanding the therapeutic arsenal for severe neurological diseases.

In addition, the translational application of integrin-targeted therapies faces many challenges. For example, the dynamic expression and diverse functions of integrins increase treatment complexity. Their expression levels and functions vary across disease stages and patients, demanding more personalized and precise therapies. Also, current drug development mainly focuses on a few integrin subtypes, with insufficient research on others, restricting the comprehensiveness and diversity of treatments. Future research should focus on several directions. First, explore integrins’ specific mechanisms in various diseases, identifying key nodes and regulatory factors in disease progression to provide a theoretical basis for precision therapy. Second, develop new integrin-targeted drugs, especially for less-studied subtypes, to expand treatment options. Third, combine cutting-edge technologies like gene editing and nanotechnology to optimize drug design and delivery systems, enhancing targeting and efficacy. Fourth, strengthen multidisciplinary cooperation, integrating knowledge and technologies from clinical medicine, biology, materials science, etc., to accelerate the clinical translation of integrin-targeted therapy.

## Therapeutic Resistance and Long-Term Efficacy of Integrin-targeted Therapies

Integrin-targeted therapies have shown significant promise in the treatment of neurological disorders such as stroke, MS, AD, and PD, where integrin signaling plays a crucial role in neuroinflammation, tissue repair, and neuronal survival. However, as with many biological therapies, therapeutic resistance may emerge over time, limiting the long-term efficacy of these treatments. The development of resistance can become a major obstacle to sustained therapeutic success (Cai et al., 2023), particularly in diseases characterized by chronic, progressive inflammation and tissue damage.

Therapeutic resistance in integrin-targeted treatments can arise through various mechanisms, often driven by intricate interactions between integrins, their ligands, and intracellular signaling pathways. In the context of neurodegenerative diseases like MS and AD, resistance often manifests through adaptive cellular responses that diminish therapeutic efficacy over time. One such mechanism involves the compensatory upregulation of alternative integrin receptors or adhesion molecules. For instance, inhibition of a specific integrin, such as β1 integrin, may prompt cells to increase the expression of other integrins, such as β3 integrin (Hamidi and Ivaska, 2018), which can bypass the therapeutic blockade, thereby compromising the treatment’s effectiveness. In MS, where neuroinflammation and immune cell migration play pivotal roles, this compensatory mechanism can reduce the overall efficacy of integrin inhibitors, underscoring the need for multitargeted strategies. Furthermore, integrins are central to several key signaling pathways, including FAK, PI3K/Akt, and MAPK, which govern cellular processes like migration, survival, and inflammation. Resistance may emerge when cells activate alternative pathways, such as NF-κB or JAK-STAT signaling, which continue neuroinflammatory responses despite integrin inhibition, further limiting the effectiveness of integrin-targeted therapies over prolonged treatment periods. Finally, immune evasion mechanisms, wherein microglia and other immune cells adapt to sustained integrin inhibition by altering their functions, may also contribute to therapeutic resistance. In AD, for example, microglial polarization to a pro-inflammatory phenotype can influence the therapeutic benefits of integrin inhibitors, despite initial success in reducing inflammation (Cai et al., 2022). These factors collectively highlight the complexity of therapeutic resistance in integrin-targeted treatments and underscore the need for novel approaches to overcome these challenges in chronic neuroinflammatory diseases.

To mitigate the potential therapeutic resistance in integrin-targeted treatments and enhance their long-term efficacy, several strategies have emerged. One promising approach is the use of combination therapies, which concurrently target multiple pathways to reduce the likelihood of resistance. By combining integrin inhibitors with immune-modulatory agents, neuroprotective compounds, or anti-inflammatory drugs, the therapeutic effects can be potentiated, particularly in diseases like MS and AD. For instance, pairing integrin inhibitors with immunosuppressive agents such as glatiramer acetate or fingolimod in MS can address various immune response aspects, minimizing compensatory mechanisms that may diminish efficacy (Candido et al., 2016). Targeted delivery systems also present a critical avenue for improving the specificity and reducing off-target effects of integrin inhibitors. Upadhyay (2014) discussed that cyclic arginine-glycine-aspartate (cRGD) binds to αVβ3 integrins, which are overexpressed in the angiogenic vasculature system and tumor cells, and is introduced into liposomes. Liposomal formulations or mAbs conjugated with small molecules can precisely target specific integrin receptors involved in processes like neuroinflammation and synaptic damage, and these systems can also be designed to bypass the BBB, a major obstacle in treating neurological disorders (Teixeira et al., 2023; Wu et al., 2023a). Biomarker-driven approaches offer another strategy by utilizing genetic and molecular profiling to predict which patients are most likely to develop resistance to integrin-targeted therapies (Morse and Gillies, 2010). By identifying specific genetic variants or the expression profiles of integrins and their downstream signaling partners, these biomarkers could guide personalized treatment adjustments, ensuring the most effective therapeutic interventions. Finally, the development of next-generation integrin inhibitors seeks to address the limitations of current therapies. These novel biologics aim to target new integrin binding sites, circumvent resistance mechanisms, and improve selectivity for integrin subtypes involved in neurodegenerative processes. By precisely targeting integrin-ligand interactions or using allosteric modulators to fine-tune integrin signaling, these inhibitors could bypass compensatory upregulation of alternative receptors, thereby improving long-term therapeutic outcomes.

Ensuring the long-term efficacy of integrin-targeted therapies in chronic neurological disorders, necessitates continuous monitoring of treatment outcomes and potential side effects. Given that integrin-based therapies influence a wide range of cellular processes, it is crucial to regularly assess their safety, efficacy, and tolerability over time. Long-term safety assessments, including biomarker monitoring, immunological evaluations, and neuroimaging, should be integrated into clinical practice to identify adverse effects or emerging resistance early. Concurrently, patient-reported outcomes—such as cognitive function, quality of life, and physical performance—should be combined with biological markers of neuroinflammation, neuronal injury, and tissue repair to gauge treatment effectiveness. These assessments are vital for guiding therapeutic adjustments, and ensuring that patients maintain optimal outcomes. Therapeutic resistance remains a significant challenge in the development of integrin-targeted therapies, arising from mechanisms such as compensatory upregulation of alternative integrins, activation of alternative signaling pathways, and immune evasion. However, strategies like combination therapies, targeted delivery systems, and biomarker-driven approaches offer promising solutions to overcome these challenges and enhance long-term efficacy. By continuously monitoring patient responses and developing novel integrin modulators, it is possible to improve the sustainability of these therapies and provide more durable outcomes for patients with chronic neurological diseases.

## Ethical and Regulatory Considerations of Integrin-targeted Therapies

Integrin-targeted therapies hold substantial promise for the treatment of neurological disorders, with the potential to achieve breakthroughs in neuroprotection, neuroinflammation, and tissue repair. However, the application of these therapies has given rise to significant ethical and regulatory concerns that necessitate careful consideration of their ethical implications, regulatory barriers, and potential long-term effects during the advancement of clinical trials.

Clinical trials involving integrin-targeted therapies must take into account the complexity of neurodegenerative diseases, the vulnerability of the patient population, and the risks associated with altering integrin signaling. Informed consent is of paramount importance, with patients needing to be fully apprised of the potential risks and benefits of participating in studies, especially the novel or unpredictable impacts on immune function, neuroinflammation, and tissue repair. For diseases predominantly affecting the elderly, such as AD and PD, it is crucial to safeguard vulnerable populations and inform patients of the systemic effects of integrin-targeted therapies, such as alterations in immune responses or BBB integrity.

Furthermore, the balance between potential benefits and risks must be carefully maintained in clinical trials, as integrins are ubiquitous in various biological processes, and their modulation may lead to unintended consequences. Additionally, it is essential to ensure the accessibility of these innovative therapies to all patients, particularly those from underrepresented or low-income groups, to reduce healthcare disparities and ensure equitable distribution of these potentially life-saving treatments.

From a regulatory standpoint, the approval of novel biologics targeting integrins, such as mAbs, peptides, and small molecule inhibitors, presents numerous challenges. As a category of biologics—complex products derived from living organisms—integrin-targeted therapies require rigorous testing for safety, efficacy, and long-term effects. Regulatory bodies demand a comprehensive evaluation of clinical trial data to ensure that these therapies are both safe and effective for their intended use. Specifically, biologics targeting integrin receptors must undergo extensive preclinical and clinical testing to assess the risks of immune responses and off-target effects.

The design of clinical trials must also consider the heterogeneity of neurological diseases and the broad impact of integrin signaling. Trial endpoints should reflect improvements in neurological function and changes in biological markers, and the duration of the trials must be sufficient to capture the potential long-term effects of integrin modulation on disease progression and overall health. Given that integrin modulation may affect the long-term functioning of organs and tissues beyond the CNS, regulatory agencies must ensure that long-term monitoring is a mandatory component of clinical trials and that post-market surveillance is conducted to identify any emerging risks.

In the context of regenerative medicine, the use of stem cell and gene therapies to manipulate integrin signaling introduces additional regulatory considerations. Stem cell therapies, including those targeting integrins to promote neurogenesis or tissue repair, face regulatory challenges such as ensuring cell purity, genetic stability, and safe engraftment in patients. Comprehensive studies are needed to understand the long-term behavior of integrin-modulated stem cells in the body, including the risks of tumor formation, immune rejection, or ectopic tissue growth. Gene therapies, which manipulate integrin gene expression through gene-editing technologies or viral vectors, must be safe, precise, and effective without causing unintended genetic alterations or immune responses. Ethical concerns surrounding gene editing and germline modifications must also be addressed.

In addition, integrin-targeted therapies have a more pronounced impact on vulnerable patient populations. Their high cost leads to uneven medical resource distribution, making it hard for economically disadvantaged groups to access these therapies. This not only limits patients’ access to optimal treatment but may also widen health disparities among different socioeconomic groups. Moreover, the underrepresentation of vulnerable populations in clinical trials means the efficacy and safety data may not accurately reflect their real-world situations. Additionally, vulnerable patients face more barriers in medical decision-making, often lacking adequate resources and opportunities to understand the therapies fully, which affects their informed consent and treatment choices. Finally, due to financial difficulties or limited access to medical resources, vulnerable patients often struggle to complete long-term follow-up, hindering comprehensive treatment-effect assessment and timely assistance when adverse events occur. Future research and practice should focus on several key areas: developing affordable treatment options by controlling costs and innovating technologically; enhancing clinical trial inclusivity by actively recruiting vulnerable groups; providing education and support to help vulnerable patients engage better in medical decision-making; and establishing monitoring and support systems to ensure these patients can complete long-term follow-up and treatment. These measures can help address the ethical challenges of integrin-targeted therapies and ensure their equitable application and safety in clinical settings.

In conclusion, the ethical and regulatory considerations surrounding integrin-targeted therapies are multifaceted and require meticulous attention as these treatments progress toward clinical application. The ethical concerns regarding patient safety, informed consent, and equitable access to treatments must be balanced with the scientific potential of integrin modulation for neurological diseases. Simultaneously, regulatory challenges associated with the approval of novel biologics, stem cell therapies, and gene therapies must be addressed through comprehensive testing, long-term monitoring, and robust regulatory frameworks. Only through careful consideration of these factors can integrin-based therapies be developed and deployed safely and effectively in clinical practice.

## Limitations

Despite advances in elucidating the pathological involvement of integrins in neurological disorders, current research faces multiple limitations. First, the heterogeneity of disease models—predominantly relying on rodent systems or oversimplified *in vitro* platforms—fails to comprehensively recapitulate the intricate interplay among genetic predisposition, environmental factors, and aging processes in multifactorial diseases such as AD and PD. Second, while acute-phase events (e.g., stroke onset) dominate research focus, longitudinal data remain scarce regarding the dynamic functional roles of integrins in chronic neuroinflammation, progressive neurodegeneration, and long-term recovery phases. Third, clinical translation remains suboptimal: aside from the success of natalizumab (anti-α4 integrin) in multiple sclerosis, most integrin-targeted therapeutics face barriers such as inadequate BBB penetration, off-target effects, and immunogenicity, hindering progression to late-stage clinical trials. Fourth, mechanistic understanding of integrin-mediated signaling networks—particularly their crosstalk with growth factor receptors, adhesion molecules (e.g., cadherins), and extracellular matrix components—remains incomplete, impeding the rational development of combination therapies targeting multiple disease pathways. To address these gaps, future studies should employ advanced methodologies such as organoid-based models, intravital imaging, and single-cell multi-omics approaches to resolve spatiotemporal integrin dynamics, coupled with biomarker-integrated clinical trial designs to fully realize the therapeutic potential of integrin modulation in neurological pathologies.

## Conclusion and Perspectives

Integrins have increasingly gained attention as key regulatory factors in the pathogenesis and progression of neurological disorders, positioning them at the forefront of neuropathological research. These molecules not only serve as essential mediators of cell adhesion but also exert bidirectional signaling functions across multiple biological processes, including inflammatory cell recruitment, angiogenesis, neurodevelopment, and neuronal survival. By orchestrating these cellular mechanisms, integrins play an indispensable role in maintaining neural homeostasis and functional integrity. Recent studies have provided compelling evidence that integrin-targeted interventions hold significant therapeutic potential in neuroprotection, inflammation modulation, BBB stabilization, and synaptic maintenance. This perspective establishes a theoretical framework for the application of integrins as therapeutic targets in neurological disorders. This review provides a comprehensive analysis of integrin functions in a spectrum of neurological diseases, including stroke (both ischemic and hemorrhagic), AD, PD, and MS. By delineating the diverse biological roles of integrins in these pathologies, we emphasize their importance as viable therapeutic targets. Integrins are not only implicated in disease initiation but may also play critical roles in disease progression, influencing key pathophysiological processes. Thus, further research and drug development aimed at integrin-based therapeutic strategies could provide novel avenues for neurological disease treatment and may emerge as a crucial component of clinical interventions.

As the crucial role of integrins in neurological diseases is progressively unveiled, future research should focus on the following key areas: First, the development of novel integrin inhibitors is essential. It is necessary to leverage computer-aided drug design technology, combined with the three-dimensional structural information of integrins, to screen for and design highly specific inhibitors, and to precisely optimize small-molecule compounds to reduce side effects on non-target tissues and achieve precise treatment. Meanwhile, the development of multi-target integrin inhibitors is also an effective strategy given the complex functions of integrins in various cellular processes. Second, the functions of integrins in neurological diseases that have not been fully investigated should be explored. Currently, research on the role of integrins in neurodevelopmental disorders (such as autism spectrum disorders and intellectual disabilities) is limited. Future research should delve into their expression patterns, functional abnormalities, and associations with neurodevelopmental processes in these diseases. For example, the impact of integrin gene mutations on brain structural and functional development can be examined by using gene knockout or knock-in animal models in combination with neuroimaging techniques. Additionally, the functions of integrins may be closely related to the stability of neuromuscular junctions and the survival of muscle cells in neuromuscular diseases (such as muscular dystrophy and peripheral neuropathies). Investigating the mechanisms of integrin action in these diseases can help develop new therapeutic strategies for neuromuscular disorders. Lastly, studying the interactions between integrins and other signaling pathways is also an important direction for future research. Neuroinflammation is one of the core pathological processes in many neurological diseases, and integrins play an important role in the recruitment and activation of inflammatory cells. However, the interaction mechanisms between integrins and neuroinflammatory signaling pathways are not yet fully understood. Future research should thoroughly investigate how integrins regulate these signaling pathways to influence the progression of neuroinflammation and how to achieve anti-inflammatory treatment by intervening in this interaction. Meanwhile, neuroplasticity is an important mechanism for the brain to adapt to environmental changes and disease injuries. Integrins play important roles in synaptic formation, remodeling, and neuronal survival, but their synergistic actions with neuroplasticity-related signaling pathways still need further investigation. By revealing the interaction networks between integrins and these signaling pathways, new strategies for promoting neuroplasticity and neural repair can be provided.

In summary, the research prospects of integrins in neurological diseases are broad. Future research should focus on developing novel integrin inhibitors, exploring the functions of integrins in neurological diseases that have not been fully investigated, and studying the interactions between integrins and other signaling pathways, thereby providing more precise and effective strategies for the treatment of neurological diseases. We anticipate that with continued advancements in this field, integrin-targeted therapeutics will drive innovation in the treatment of neurological disorders, ultimately improving patient outcomes and quality of life.

## Additional files:

***Additional file 1:*** *Open peer review report 1.*

OPEN PEER REVIEW REPORT 1

***Additional Table 1:*** *The research trajectory of integrins.*

***Additional Table 2:*** *Classification of integrins and their functions.*

***Additional Table 3:*** *Distribution and role of integrins in the brain.*

***Additional Table 4:*** *Integrin-mediated “outside-in” and “inside-out” signaling.*

***Additional Table 5:*** *Detailed mechanisms of integrin-mediated signaling.*

***Additional Table 6:*** *Molecular crosstalk between integrins and other pathways in neurological diseases.*

***Additional Table 7:*** *Effects of integrins on neurodevelopment.*

***Additional Table 8:*** *The role of integrins in various neurological disorders.*

## Data Availability

*The data are available from the corresponding author on reasonable request.*
