# Supplementary material for: The potential and therapeutic advances of the integrin family in neurological disorders
Source: Neural Regen Res. 2025 Aug 13;21(9):4177–94. doi: 10.4103/NRR.NRR-D-25-00020 (PMC13557648; doi:10.4103/NRR.NRR-D-25-00020)
Supplement: Supplementary file 1 [file NRR-21-4177_Suppl1.pdf]

OPEN PEER REVIEW REPORT 1

Name of journal: Neural Regeneration Research
Manuscript NO: NRR-D-25-00020
Title: Potential and therapeutic advances of the integrin family in ischemic stroke: a review
Reviewer's Name: Shun Li
Reviewer's country: USA

COMMENTS TO AUTHORS

This review offers an extensive examination of integrin structure and classification, and their physiological and pathological functions in the central nervous system, emphasizing their molecular mechanisms in neurological illnesses. Additionally, the authors assess the therapeutic potential and obstacles related to integrin-targeted therapies. This review seeks to clarify the molecular foundations of integrin action in the CNS, thereby enhancing comprehension of their translational potential and establishing a basis for the creation of novel therapeutic techniques. Therefore, this review is interesting enough to merit publication here. However, the paper seems unprepared for publishing. Several details need improvement.

1. The legend only has a title. Add some description to make the explanation clearer.
2. Abbreviations should be given in full only when they appear for the first time. The author repeated the full name of BBB in lines 123 and 887. Please browse the full text to check for other similar issues. And after a term has been abbreviated, it is better to use the abbreviation exclusively
